# HI-511 overcomes melanoma drug resistance *via* targeting AURKB and BRAF V600E

**DOI:** 10.7150/thno.44342

**Published:** 2020-08-01

**Authors:** Xiaoyu Chang, Tianshun Zhang, Qiushi Wang, Moeez Ghani Rathore, Kanamata Reddy, Hanyong Chen, Seung Ho Shin, Wei-ya Ma, Ann M Bode, Zigang Dong

**Affiliations:** 1 The Hormel Institute, University of Minnesota, 801 16th Ave NE, Austin, MN 55912, USA.; 2 Program in Bioinformatics and Computational Biology, University of Minnesota, Minneapolis, MN 55455, USA.

**Keywords:** HI-511, AURKB, BRAF V600E, vemurafenib-resistant melanoma, melanoma

## Abstract

**Rationale:** Melanoma is an aggressive tumor of the skin and drug resistance is still a major problem in melanoma therapy. Novel targets and effective agents to overcome drug resistant melanoma are urgently needed in clinical therapy.

**Methods:** Gene Expression Omnibus (GEO) database analysis, pathway enrichment analysis, and survival rate analysis were utilized to identify a candidate target. An anchorage-independent cell growth assay, flow cytometry, Western blot, and a xenograft mouse model were used to study the function of Aurora kinase B (AURKB) in both drug-sensitive and drug-resistant melanoma. Next, HI-511, a novel dual-target inhibitor targeting both AURKB and BRAF V600E, was designed and examined by an *in vitro* kinase assay. Methods as indicated above in addition to a BRAF V600E/PTEN-loss melanoma mouse model were used to demonstrate the effect of HI-511 on melanoma development *in vitro* and *in vivo*.

**Results:** AURKB is highly expressed in melanoma and especially in vemurafenib-resistant melanoma and the expression was correlated with patient survival rate. Knocking down *AURKB* inhibited cell growth and induced apoptosis in melanoma, which was associated with the BRAF/MEK/ERKs and PI3-K/AKT signaling pathways. Importantly, we found that HI-511, a novel dual-target inhibitor against AURKB and BRAF V600E, suppresses both vemurafenib-sensitive and vemurafenib-resistant melanoma growth *in vitro* and *in vivo* by inducing apoptosis and mediating the inhibition of the BRAF/MEK/ERKs and PI3K/AKT signaling pathways.

**Conclusion:** AURKB is a potential target for melanoma treatment. HI-511, a novel dual-target inhibitor against both AURKB and BRAF V600E, could achieve durable suppression of melanoma growth, even drug-resistant melanoma growth.

## Introduction

Malignant melanoma is one of the most aggressive forms of skin cancer 1-3. The American Cancer Society estimated that 96,480 new melanoma cases will be diagnosed and about 7,230 people are expected to die from the disease in the United States in 2019. Acquired resistance limits chemotherapy in melanoma, leading to disease progression 4. Thus, a novel target for chemotherapy is urgently needed.

The mechanisms of melanoma development and drug resistance have been identified 4, 5. Mutations in the *BRAF* gene are the most common mutation associated with melanoma, and among such mutations, BRAF V600E has been detected in about 50% of melanoma patients 6, 7. Although BRAF V600E inhibitors, such as vemurafenib 8, are commonly used currently in melanoma treatment 9, chemotherapy is often unsuccessful due to drug resistance 10, 11, which develops after chemotherapy, rendering the patient unresponsive to the treatment. Resistance to vemurafenib occurs after treatment for 2-18 months 12. In studies of the mechanism of melanoma drug resistance, the reactivation of the BRAF/MEK/ERKs pathway is seen in about 80% of resistant tumors 13. On the other hand, reactivation of the PI3-K/AKT pathway, which interacts with the BRAF/MEK/ERKs pathway 13, 14 is another cause of drug resistance.

To overcome drug resistance in melanoma, a therapeutic approach with a combination of targets is an available strategy and has been approved by the FDA. A combination of a MEK inhibitor with a mutant BRAF inhibitor effectively increased the melanoma survival rate in a randomized clinical trial 15-17. Co-targeting melanoma with BRAF/MEK and PI3-K inhibitors to overcome BRAF inhibitor- resistance provided further evidence showing that a combination targeted therapy is necessary for melanoma treatment 18-20. In order to identify a new target, we analyzed the Gene Expression Omnibus database and selected *Aurora kinase B (AURKB)*. AURKB is a serine/threonine protein kinase 21, which is essential for a chromosome passenger protein required for phosphorylation of histone H3, chromosome segregation and cytokinesis 22. AURKB could mediate mitotic chromosome condensation by phosphorylating histone H3 at serine 10 23, inhibition of AURKB will lead to proliferation stop even cell death by apoptosis 24. In previous study, it's found that *AURKB* knockout mice (-/-) die at the blastocyst embryonic stage 25. *AURKB* is overexpressed in a wide range of cancer types 26-28. Previous studies have indicated that inhibition of AURKB can enhance radiosensitivity in androgen blockade-resistant prostate cancer 29 and suppresses the growth of cetuximab-resistant head and neck squamous cell carcinoma (HNSCC) cells 30.

In this study, we examined the function of AURKB in the development of both vemurafenib-sensitive and vemurafenib-resistant melanoma. We attempted to identify the mechanism by which AURKB could mediate drug-sensitive and drug-resistant melanoma. We also demonstrated that HI-511, a novel AURKB and BRAF V600E inhibitor, suppresses tumor growth and overcomes vemurafenib resistance *in vitro* and *in vivo*. Our findings suggest that AURKB could be a potential target for melanoma treatment.

## Materials and Methods

### Reagents and antibodies

HI-511 and APIO-EE-09 31 were synthesized and the structure of HI-511 was confirmed by NMR spectroscopy. Tris, NaCl, and SDS for molecular biology and buffer preparation were purchased from Sigma-Aldrich (St. Louis, MO). All cell culture media were purchased from Invitrogen (Waltham, MA). Antibodies to detect cleaved PARP (#5625), cleaved caspase-3 (#9664), p-histone H3 (#9701), histone H3 (#9715), p-BRAF (#2696), p-MEK (#9121), MEK (9122), p-ERKs (#9101), ERKs (#9102), p-PI3-K (#4228), PI3-K (#3011), p-AKT (#9271), AKT (#9272), PCNA (D3H8P; #13110) were obtained from Cell Signaling Technology (Danvers, MA). β-Actin (sc-47778), PARP-1 (sc-74470), caspase-3 (sc-7272), Bcl-2 (sc-7382), Bax (sc-20067), and GAPDH (sc-32233) antibodies were from Santa Cruz Biotechnology (Dallas, TX). BRAF (07-583) was from MilliporeSigma (Burlington, MA), AURKB (NB100-294) was from Novus Biologicals (Centennial CO), and vemurafenib was purchased from Cayman Chemical Co. (Ann Arbor, MI).

### Cell culture and transfection

The A375 and SK-MEL-31 melanoma cell lines were obtained from the American Type Culture Collection (ATCC) and maintained following ATCC instructions. Normal human epidermal melanocytes (NHEM) were purchased from PromoCell (Heidelberg, Germany). A375 cells were cultured in DMEM containing 10% FBS and 1% antibiotics. Cells were cytogenetically tested and authenticated before being frozen. SK-MEL-31 cells were cultured in MEM containing 15% FBS and 1% antibiotics with 2 mM _L_-glutamine and Earle's BSS adjusted to contain 1.5 g/L sodium bicarbonate, 0.1 mM non-essential amino acids, and 1.0 mM sodium pyruvate. Each vial of frozen cells was thawed and maintained for a maximum of 20 passages. To develop a vemurafenib-resistant A375 cell line, cells were treated with a 1 μM concentration of vemurafenib every 3 days for 4-6 weeks. The concentration of vemurafenib was then increased up to 2 μM and cells were treated every 3 days for 4-6 weeks. The vemurafenib concentration was increased up to 5 μM and cells were treated with vemurafenib at 5 μM twice a week to maintain drug resistance 1, 32.

The M238, M238R, M249, and M249R melanoma cell lines were obtained from the University of California, Los Angeles 32. M238 and M249 cells were cultured in DMEM containing 10% FBS and 1% antibiotics. The M238 and M249 melanoma cell lines are BRAF V600E-positive, and the M238R and M249R melanoma cell lines are vemurafenib-resistant sub-lines 33. To maintain the vemurafenib-resistant M238R and M249R cell lines, cells were treated with 1 μM vemurafenib every 3 days. Then the concentration of vemurafenib was increased up to 2 μM and cells were treated every 3 days for 4-6 weeks. The vemurafenib concentration was increased to 5 μM and cells were treated with vemurafenib at 5 μM twice a week to maintain drug resistance 1.

For lentiviral transfection, the jetPEI reagent (Qbiogene, Inc., Montreal, Quebec, Canada) was used following the manufacturer's instructions. The 29-mer small hairpin RNA (shRNA) constructs were against human and the lentivirus plasmids shAURKB (#1, TRCN0000000778; 5'-TGATGGAGAATAGCAGTGGGA-3', #2 TRCN0000010547; 5'-GCATCACACAACGAGACCTAT-3') were from the University of Minnesota Genomic Center (University of Minnesota, Minneapolis, MN). The pLKO.1-puro Non-Target shRNA Control Plasmid DNA (shcontrol) was from Sigma-Aldrich (St. Louis, MO).

### Anchorage-independent growth assay

In each well of a 6-well plate, cells (8 × 10^3^) were suspended in Basal Medium Eagle (BME) medium (1 mL, with 10% FBS and 0.33% agar) and plated over a layer of solidified BME (3 mL, with 10% FBS and 0.5% agar). The cultures were incubated in a 37 °C, 5% CO_2_ incubator for 1-2 weeks and then colonies in soft agar were counted under a microscope equipped with the Image-Pro Plus software program (Media Cybernetics, Bethesda, MD).

### Flow cytometry for apoptosis analysis and cell cycle

Flow cytometry was used for analysis of apoptosis and cell cycle. All the shcontrol and knockdown groups of A375, A375R, M249, and M249R cells were seeded (2.5×10^5^/well) into 60-mm dishes. The cells were incubated for 48 h and then washed twice with cold phosphate-buffered saline (PBS), resuspended with PBS and incubated for 5 min at room temperature with annexin V-FITC and propidium iodide. Samples were analyzed using a FACSCalibur flow cytometer (BD Biosciences, San Jose, CA). For treatment, A375, A375R, M249, and M249R cells (2.5×10^5^/well) were seeded into 60-mm dishes. Cells were incubated overnight, and on the second day, cells were treated with DMSO or HI-511 for 48 h. Cells were washed twice with cold PBS and then resuspended with PBS and incubated for 5 min at room temperature with annexin V-FITC and propidium iodide. Samples were analyzed using a FACSCalibur flow cytometer (BD Biosciences, San Jose, CA). The shcontrol and shAURKB transfected A375 or A375R cells were established and subjected to cell cycle assay. The cells (2.5×10^5^/well) were seeded into 60-mm dishes and incubated for 24 h. To study the effects of HI-511 on the cell cycle distribution of A375 or A375R, the cells were treated with HI-511 or DMSO for 24 h. After washing twice with cold PBS and fixed in cold 70% ethanol for 30 min. Staining by using propidium iodide and samples were analyzed using a FACSCalibur flow cytometer.

### Western blot analysis

Protein samples (30 μg) were resolved by SDS-PAGE and transferred to Hybond C nitrocellulose membranes (Amersham Corporation, Arlington Heights, IL). After blocking with 5% fat-free milk for 1 h, the membranes were incubated with primary antibodies (1:1000) overnight at 4 °C. The targeted protein bands were visualized using an enhanced chemiluminescence reagent (Amersham Corporation) after hybridization with a secondary antibody conjugated with horseradish peroxidase.

### Enzyme-linked immunosorbent assay (ELISA)

The epidermal growth factor (EGF) level in xenograft tumor sample was measured by the human EGF ELISA kit (Sigma-Aldrich, Inc). The protein samples were isolated following the ELISA instruction. Briefly, 100 μL of each sample was added into appropriate wells and incubated for 2.5 h at room temperature with shaking. After washing four times, 100 μL of Biotinylated Detection Antibody was incubated for 1 h at room temperature with shaking. After washing four times, 100 μL of HRP-Streptavidin solution was incubated for 45 min at room temperature with shaking. After washing four times, 100 μL of ELISA Colorimetric TMB Reagent was incubated for 30 min at room temperature with shaking. Then, the stop solution (Item I, 50 μL) was added to each well and read at 450 nm immediately.

### Crystal violet staining assay

The cells (2 × 10^4^) were seeded into 24-well plate and incubated in a 37 °C, 5% CO_2_ incubator for 24 h. Then the cells were treated with compounds for 48 h. Next, the cells were washed three times with distilled water and stained with 0.2% (w/v) crystal violet in 2% (v/v) ethanol-water for 5 min. Cells were washed three times with distilled water again and dry. After took photos of plate, the stained dye was dissolved in 0.5% (w/v) SDS in 50% (v/v) ethanol-water. Finally, measuring the absorbance at 540 nm wavelength.

### Gene set enrichment analysis (GSEA)

GSEA was performed by GSEA v4.0.2 for Windows (Broad Institute, MIT, USA). The expression dataset was downloaded from GEO (GSE: 4587) and the gene set “c2.cp.kegg.v6.0.symbols.gmt” was used in analysis. The number of permutations was 1000 and the phenotype label is *AURKB* high and low expression.

### Computational docking

Computer modeling of HI-511 with AURKB and BRAF V600E was performed using the Schrödinger Suite 2018 software programs 34. The protein structures of AURKB (PDB: 4AF3) 35, BRAF V600E (PDB: 3PPJ) and wild-type BRAF (PDB:1UWH) 36 were downloaded from the Protein Data Bank (PDB). The AURKB and BRAF V600E crystal structures were prepared using the standard procedure of the Protein Preparation Wizard in Schrödinger Suite 2018. Hydrogen atoms were added consistent with a pH of 7 and all water molecules were removed. The ATP binding site-based receptor grid was generated for docking. HI-511 was prepared using the LigPrep program (Schrödinger) and the lowest energy conformations for docking were determined by using default parameters under the extra precision (XP) mode and the Glide program. The protein-ligand docking analysis was conducted using the induced fit docking program of Schrödinger, which can provide ligand binding flexibility with binding pocket residues.

### MTS assay

Melanocyte cells (1×10^4^ cells/well) were seeded into 96-well plates in 100 mL of medium. After 24 h of culture, the appropriate concentration of each compound was added to each well. After incubation for another 24 h or 48 h, 20 μL of the CellTiter 96 Aqueous One Solution (Promega Corporation, Fitchburg, WI) were added to each well and cells were incubated for an additional 1 h. Absorbance was measured at 492 nm using the Thermo Multiskan plate-reader (Thermo Fisher Scientific, Waltham, MA).

### *In vitro* kinase assays

#### *In vitro* AURKB activation kinase assay

Inactive histone H3 proteins (1 μg) were used as substrates for an *in vitro* kinase assay with 100 ng of active AURKB kinase. The concentrations of HI-511 used were 0, 1, 5 and 10 μM. A positive control group was added with the AURKB inhibitor, APIO-EE-9, which was previously developed in our laboratory 31. Reactions were conducted in 1 × kinase buffer (40 mM MOPS/NaOH pH 7.0, 1 mM EDTA, 10 mM MnCl_2_, and 0.8 M ammonium sulfate) containing 100 µM ATP and incubated at 30°C for 30 min. Reactions were terminated by addition of 10 μL protein loading buffer and the reaction products were detected by Western blot analysis 37.

#### *In vitro* mutant BRAF V600E and wild-type BRAF activation kinase assays

Inactive MEK proteins (1 μg) were used as the substrates in *in vitro* kinase assays with 100 ng of active mutant BRAF V600E and wild-type BRAF. For each reaction, the HI-511 concentrations were 0, 1, 5, and 10 μM. A positive control group for mutant BRAF V600E was added using the mutant BRAF V600E inhibitor, vemurafenib. Reactions were conducted in 1×kinase buffer (40 mM MOPS/NaOH pH 7.0, 1 mM EDTA, 10 mM MnCl2, and 0.8 M ammonium sulfate) containing 100 µM ATP and incubated at 30 °C for 30 min. Reactions were terminated by addition of 10 μL protein loading buffer and the reaction products were detected by Western blot analysis 37.

### Animal studies

All studies were performed following guidelines approved by the University of Minnesota Institutional Animal Care and Use Committee (Minneapolis, MN). The xenograft mouse model (Protocol ID: 1803-35739A) was conducted to examine the effect of knocking down expression of *AURKB* in melanoma. Athymic nude mice (6-week-old mice; Charles River Laboratories, Wilmington, MA) were inoculated in the right flank with shcontrol- or shAURKB-expressing melanoma cells (A375, A375R, M249, or M249R cells, 2×10^6^ cells/mouse; n = 6).

Another xenograft mouse model (Protocol ID: 1803-35739A) was conducted to study the effect of HI-511 on melanoma tumor growth. Athymic nude mice (6-week-old mice; Harlan Laboratory, IN) were inoculated in the right flank with vemurafenib-sensitive A375 (1.5×10^6^ cells/mouse, n = 24) or M249 cells (2×10^6^ cells/mouse, n = 24) and the left flank with vemurafenib-resistant A375R (1.5×10^6^ cells/mouse) or M249R cells (2×10^6^ cells/mouse).To study the effect of combination of HI-511 and vemurafenib on vemurafenib-resistant melanoma tumor growth, A375R (2×10^6^ cells/mouse) cells were injected in the right flank of athymic nude mice. Then the mice were randomly divided into four groups (n = 6 in each group) as follows: 1) vehicle group; 2) treatment with 50 mg/kg vemurafenib; 3) treatment with 50 mg/kg vemurafenib and 10 mg/kg HI-511 and 4) treatment with 50 mg/kg vemurafenib and 50 mg/kg HI-511. Treatment was administered by oral gavage. Mice were maintained under "specific pathogen-free" conditions based on the guidelines established by the University of Minnesota Institutional Animal Care and Use Committee. Mice were randomly divided into four groups (n = 6 in each group) as follows: 1) vehicle group; 2) treatment with 10 mg/kg HI-511; 3) treatment with 50 mg/kg HI-511; and 4) treatment with 50 mg/kg vemurafenib as a control. Treatment was administered by oral gavage. Tumor volume and body weight were measured once a week. HI-511 was prepared in PBS with 2.5% DMSO, 5% polyethylene glycol 400 (PGE 400), and 5% tween 80. Tumor volume was calculated from 2 diameters of the individual tumor base using the following formula: tumor volume (mm^3^) = length × width × width × 0.52. This *BARF* mutant/ *PTEN*-null/cre mouse model (Protocol ID: 1805-35962A) was used to confirm the effect of HI-511 on tumor growth. *BRAF V600E*/ *PTEN*-null mice (B6. Cg-Braftm1Mmcm Ptentm1Hwu Tg(Tyr-cre/ERT2)13Bos/BosJ) were purchased from the Jackson Laboratory. The mice were housed and bred in a virus and antigen-free room. Mice were genotyped by standard PCR analysis according to the Jackson Laboratory genotyping protocol. Mice with *BRAF V600E* mutation heterozygote and *PTEN* loss with Cre were used in this study. For localized melanoma induction on the dorsal skin, adult mice (6-8 weeks of age) were treated topically with 2.5 μL of 1.9 mg/mL (5 mM) 4-hydroxytamoxifen (4-HT) (Sigma-Aldrich, H6278, St. Louis, MO) for 3 days. Mice were randomly divided into 3 groups (n = 8 in each group) as follows: 1) vehicle group; 2) treatment with 10 mg/kg HI-511; and 3) treatment with 50 mg/kg HI-511. HI-511 were dissolved in PBS with 2.5% DMSO, 5% PEG 400, and 5% Tween-80. The compounds were administered to mice by oral gavage and the relevant solvent was administered to control animals. The compounds or solvent were administered to the mice daily beginning at day 23, when the animals had measurable melanoma lesions. Tumor volume and body weight were measured once a week. Tumor volume was calculated from 2 diameters of the individual tumor base using the following formula: tumor volume (mm^3^) = length × width × width × 0.52.

### Immunohistochemical analysis of tissue array and mouse melanoma tissues

A human melanoma tissue array (ME803b) was purchased from the US Biomax Inc cancer tissue bank collection (US Biomax Inc, MD). A Vectastain Elite ABC Kit obtained from Vector Laboratories (Burlingame, CA) was used for immunohistochemical staining according to the protocol described by the manufacturer. Mouse melanoma tissues were fixed in 10% Buffered Formalin Phosphate (Fisher Chemical, Hampton, NH) and embedded in paraffin for examination. Sections were stained and analyzed by immunohistochemistry. Briefly, all specimens were maintained at 60 °C for 2 h, deparaffinized and rehydrated. To expose antigens, samples were unmasked by submerging each into boiling sodium citrate buffer (10 mM, pH 6.0) for 10 min, and then treated with 3% H_2_O_2_ for 10 min. The slides were blocked with 50% goat serum albumin in 1 × PBS in a humidified chamber for 1 h at room temperature. The tissue sections were hybridized with a PCNA (1:3000), c-caspase 3 (1:50), c-PARP (1:50) or a Bcl-2 (1:50) antibody at 4°C in a humidified chamber overnight. The slides were washed and hybridized with the secondary antibody from Vector Laboratories (anti-rabbit 1:200 or anti-mouse 1:200) for 1 h at room temperature. Slides were stained using the Vectastain Elite ABC Kit. After development with 3,3'-diaminobenzidine, the sections were counterstained with hematoxylin and observed by microscope (× 200) and analyzed by Image-Pro PLUS (v.6) computer software program (Media Cybernetics, Inc. Rockville, MD).

### Statistical analysis

All quantitative data are expressed as mean values ± standard error (S.E.) from at least 3 independent experiments. Significant differences were determined by parametric analysis including a two-tailed Student's* t* test and one-way ANOVA. A probability value of *p* < 0.05 was used as the criterion for statistical significance.

## Results

### AURKB could be a potential target for melanoma treatment

**Figure 1:**
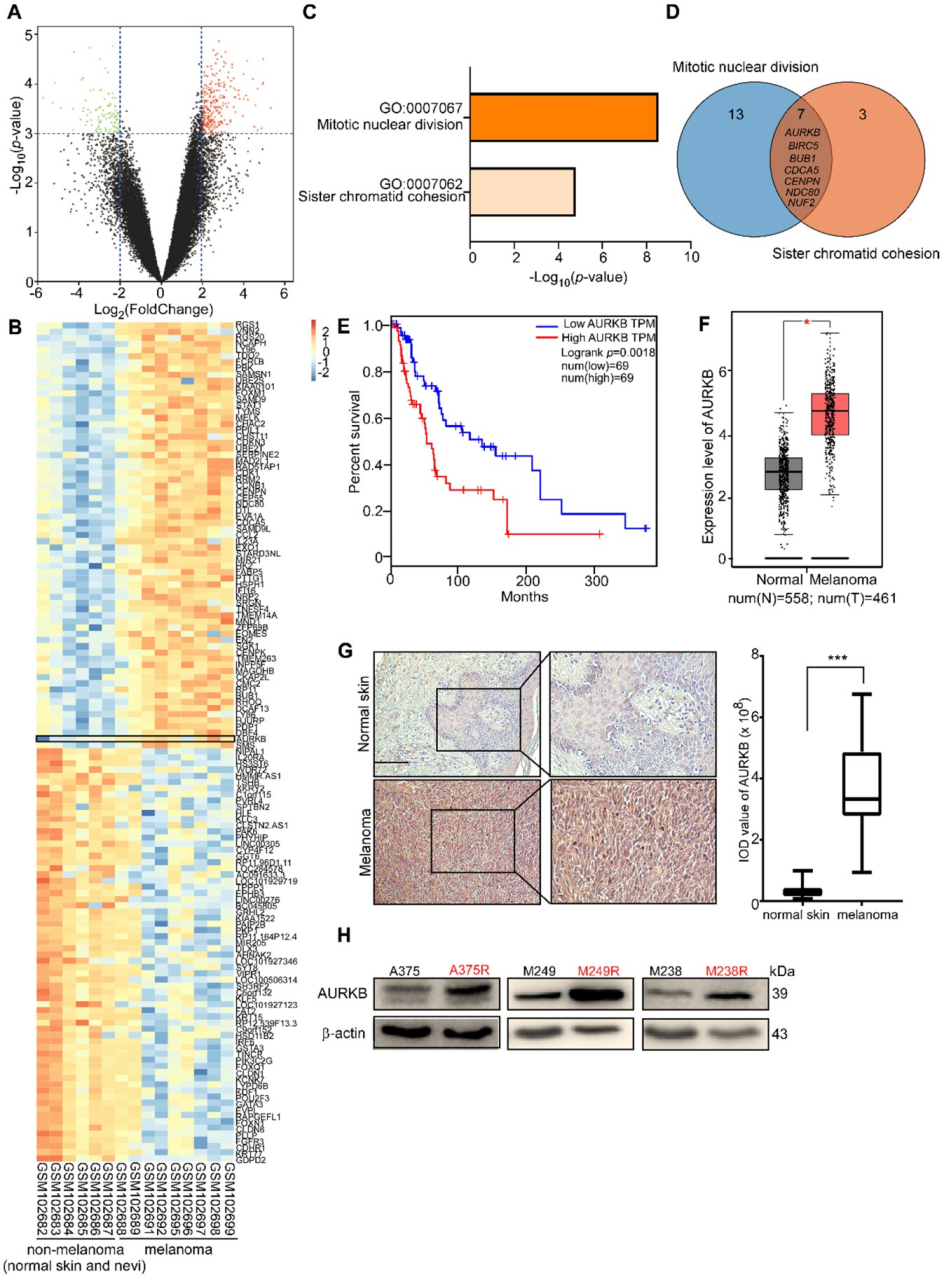
** Database analysis and *AURKB* expression level in melanoma**. **(A)** Volcano plot of gene expression analysis in GSE 4587. The X-axis indicates the fold change between normal skin or nevi and melanoma samples, and the Y-axis indicates on a log_10_ scale the *p*-values obtained from a supervised logistic regression analysis testing the association of gene expression between normal skin or nevi/melanoma. The horizontal dotted lines mark the significance cutoffs (i.e., fold change more than 4 times or less than 0.25, and *p*-value less than 0.001). **(B)** Heat map of analysis of the expression of these selected genes in GSE 4587. *AURKB* has a higher expression level in melanoma compared with non-melanoma (normal skin and nevi). **(C)** Pathway enrichment analysis of differential gene analysis. The top 2 pathways were selected, fold change more than 4 times, *p*-value less than 0.0001.** (D)** The Venn diagram lists the common genes in both pathways.** (E)** Melanoma patients with high expression of *AURKB* show a significantly lower overall survival rate.** (F)** *AURKB* is highly expressed in melanoma compared with normal skin as determined by GEPIA.** (G)** AURKB is significantly overexpressed in melanoma tissue compared with normal skin as shown in a tissue array analysis; the scale bar = 100 µm. **(H)** AURKB is overexpressed in drug-resistant melanoma cell lines (A375R, M238R, and M249R) compared with drug-sensitive melanoma cell lines (A375, M238 and M249). Statistical significance was determined by Student's *t*-test and the asterisks indicate a significant change compared with the control group (*, *p* < 0.05; ***, *p* < 0.001).

By analyzing the available data from the GEO database (GSE: 4587), we found differential levels of gene expression between non-melanoma (normal skin, nevi) and melanoma (**Figure 1A**). The significantly different expression levels of genes (i.e., fold change more than 4 or less than 0.25; *p*-value less than 0.001) between non-melanoma (normal skin, nevi) and melanoma are shown in a heat map (**Figure 1B**). Enrichment of the signaling pathway analysis was performed using DAVID 38, 39, and the Gene Ontology (GO) results revealed that the genes were significantly enriched in the mitotic nuclear division pathway and the sister chromatid cohesion pathway (**Figure 1C**). The candidate/shared genes (*AURKB*, *BIRC5*, *BUB1*, *CDCA5*, *CENPN*, *NDC80,* and *NUF2*) were identified from a two-way Venn diagram (i.e., the mitotic nuclear division pathway and sister chromatid cohesion pathway) (**Figure 1D**). The overall survival rate was obtained from the human skin cutaneous melanoma (SKCM) sample data taken from the TCGA and the GTEx projects 40. The probability of survival of patients with higher *AURKB* expression is significantly lower than that of patients with lower *AURKB* expression (**Figure 1E**). However, the probability of survival of patients did not show a significant difference in expression of the other 6 candidate genes (i.e., *BIRC5*, *BUB1*, *CDCA5*, *CENPN*, *NDC80,* or *NUF2*; **Figure S1**). Additionally, *AURKB* is expressed significantly higher in melanoma tissues compared with normal tissues (normal, n = 558, tumor, n = 461; **Figure 1F**). Immunohistochemical (IHC) analysis of the AURKB expression level in the normal skin and melanoma tissue array revealed that AURKB was overexpressed and significantly higher in melanoma (**Figure 1G**). Notably, AURKB is expressed markedly higher in vemurafenib-resistant (A375R, M238R, and M249R) compared with vemurafenib-sensitive melanoma cell lines (A375, M238, and M249; **Figure 1H**). All these results demonstrate that AURKB could be a potential target for melanoma treatment and in overcoming drug resistance.

### AURKB is crucial for melanoma proliferation, apoptosis and cell cycle

**Figure 2:**
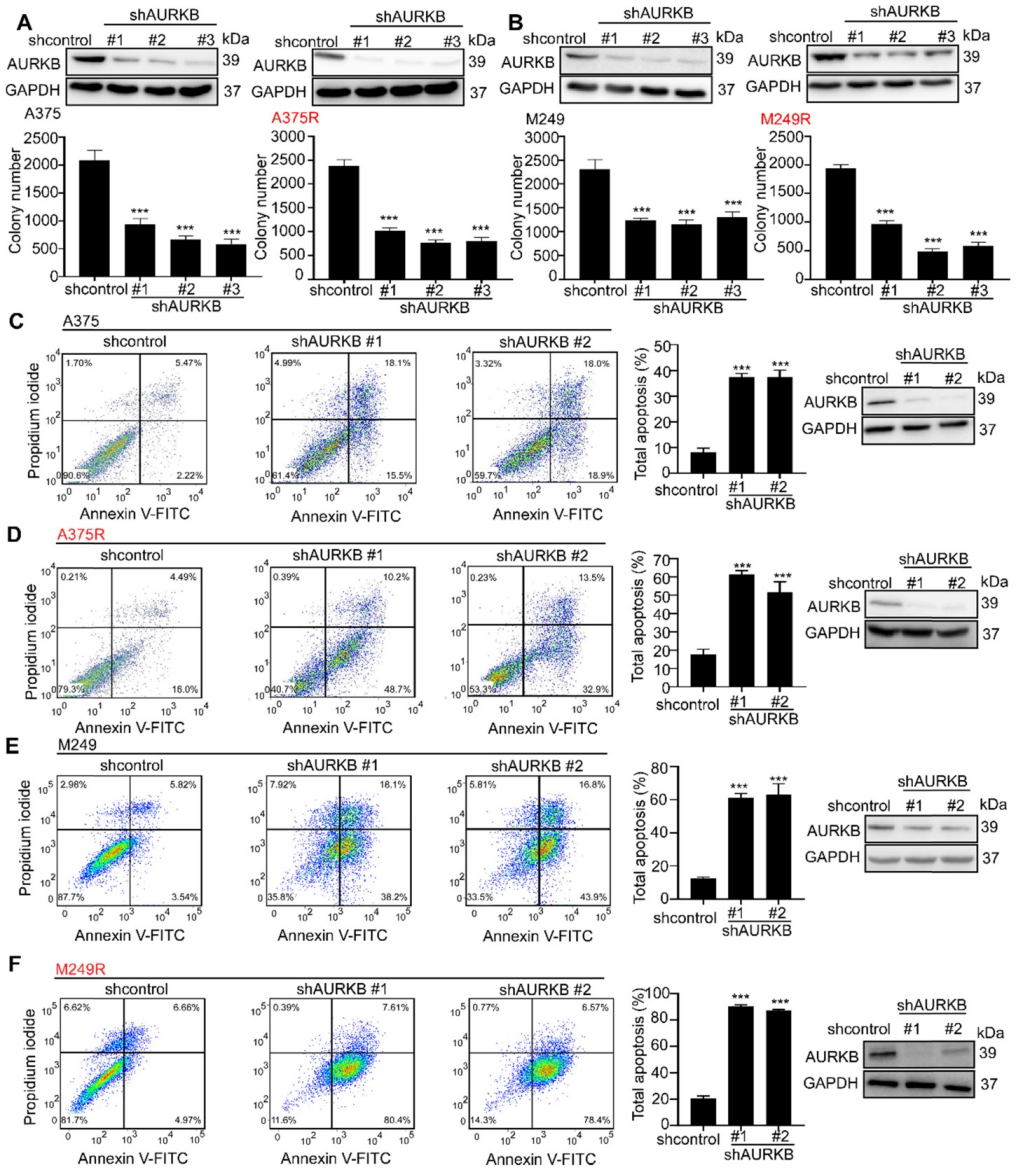
** Knocking down expression of *AURKB* mediates growth and apoptosis in melanoma cells.** A375, A375R, M249, and M249R melanoma cells with stable knockdown of *AURKB* were established. **(A)** Knocking down *AURKB* suppresses A375 and A375R cell growth as determined by an anchorage-independent growth assay. **(B)** Knocking down expression of *AURKB* suppresses M249 and M249R cell growth as determined by an anchorage-independent growth assay. Knocking down expression of *AURKB* induced **(C)** A375, **(D)** A375R,** (E)** M249 and **(F)** M249R apoptosis rate as shown by flow cytometry analysis. Statistical significance was determined by one-way ANOVA and the asterisks indicate a significant change compared with the control group (***, *p* < 0.001).

In cell-based studies, we transfected shRNA lentivirus to knock down expression of *AURKB* in both vemurafenib-sensitive (A375 and M249) and vemurafenib- resistant melanoma cell lines (A375R and M249R). The expression level of *AURKB* was validated by Western blot. Our results indicated that knocking down the expression of *AURKB* could induce G2/M arrest in both A375 and A375R (**Figures S2A, B**). Furthermore, we found that knocking down *AURKB* significantly suppressed melanoma cell growth in an anchorage-independent growth assay (**Figures 2A, B**). Flow cytometry results revealed that knocking down the expression of *AURKB* induced apoptosis in both vemurafenib-sensitive and -resistant melanoma cell lines (**Figures 2C-F**).

### Knocking down *AURKB* suppresses melanoma growth in a xenograft mouse model

**Figure 3:**
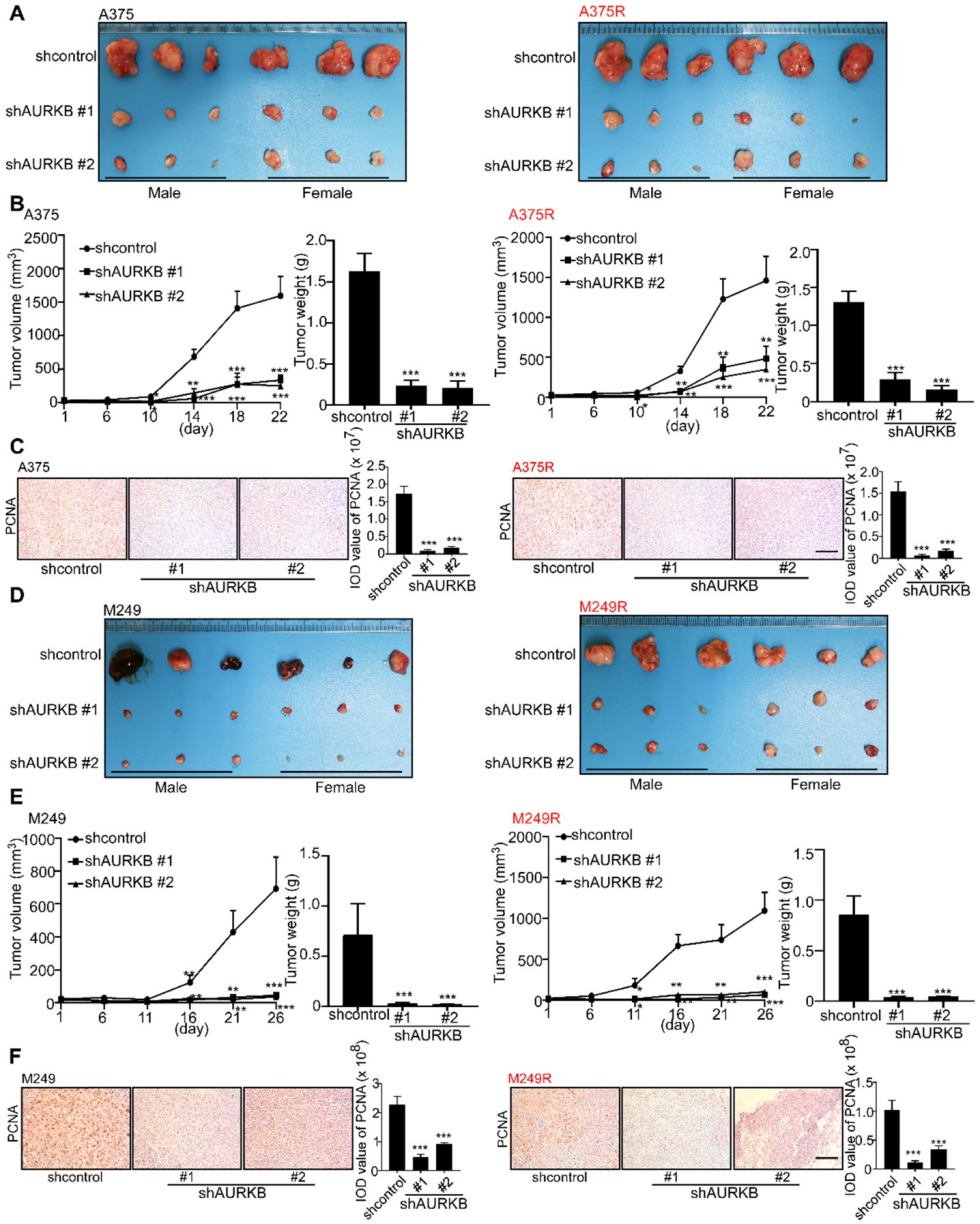
** Knockdown of *AURKB* decreases tumor growth in a xenograft mouse model. (A)** Tumors from the A375 and A375R xenograft models. **(B)** Tumor size in each group was measured every week and tumor weight was measured at day 22. **(C)** The expression of PCNA in A375 and A375R tumors in the xenograft model was detected by immunohistochemistry staining; the scale bar = 100 µm. **(D)** Tumors from M249 and M249R xenograft models. **(E)** Tumor size in each group was measured every week and tumor weight was measured at day 22. **(F)** The expression of PCNA in M249 and M249R tumors from the xenograft model was detected by using immunohistochemistry staining; the scale bar = 100 µm. Statistical significance was determined by one-way ANOVA. The asterisks indicate a significant difference compared with the shcontrol group (***, *p* < 0.001).

A xenograft mouse experiment was conducted to determine the effect of *AURKB* on melanoma tumor growth *in vivo*. At 22 days after tumor cell injection, the tumors with partial silenced *AURKB* were diminished compared with the scramble control tumors (**Figure 3A**). The changes in tumor volume and tumor weight confirmed a significant reduction in tumors with partial silenced *AURKB* in both vemurafenib-sensitive (A375) and -resistant melanoma (A375R; **Figure 3B**). Immunohistochemical data showed that the expression of the proliferating cell nuclear antigen (PCNA) was significantly attenuated by knocking down *AURKB* (**Figure 3C**). Next, we injected another vemurafenib-sensitive (M249) or -resistant melanoma cell line (M249R) with or without partial silencing of *AURKB* into this xenograft mouse model. Tumor images are shown (**Figure 3D**) and knocking down *AURKB* significantly reduced both tumor volume and weight (**Figure 3E**). Furthermore, significantly decreased PCNA expression level was observed in tumors expressing knock down *AURKB* (**Figure 3F**). The expression of c-caspase 3 and c-PARP in tumors was detected by IHC. The results showed that the apoptosis level was significantly increased by knocking down *AURKB* compared with shcontrol group in both vemurafenib-sensitive (A375, M249) and -resistant melanoma (A375R, M249R) cell lines (**Figures S3A-D**). All these results indicate that AURKB could be a potential target for melanoma treatment and in overcoming drug resistance.

### AURKB could mediate the BRAF/MEK/ERKs and PI3-K/AKT pathways

**Figure 4:**
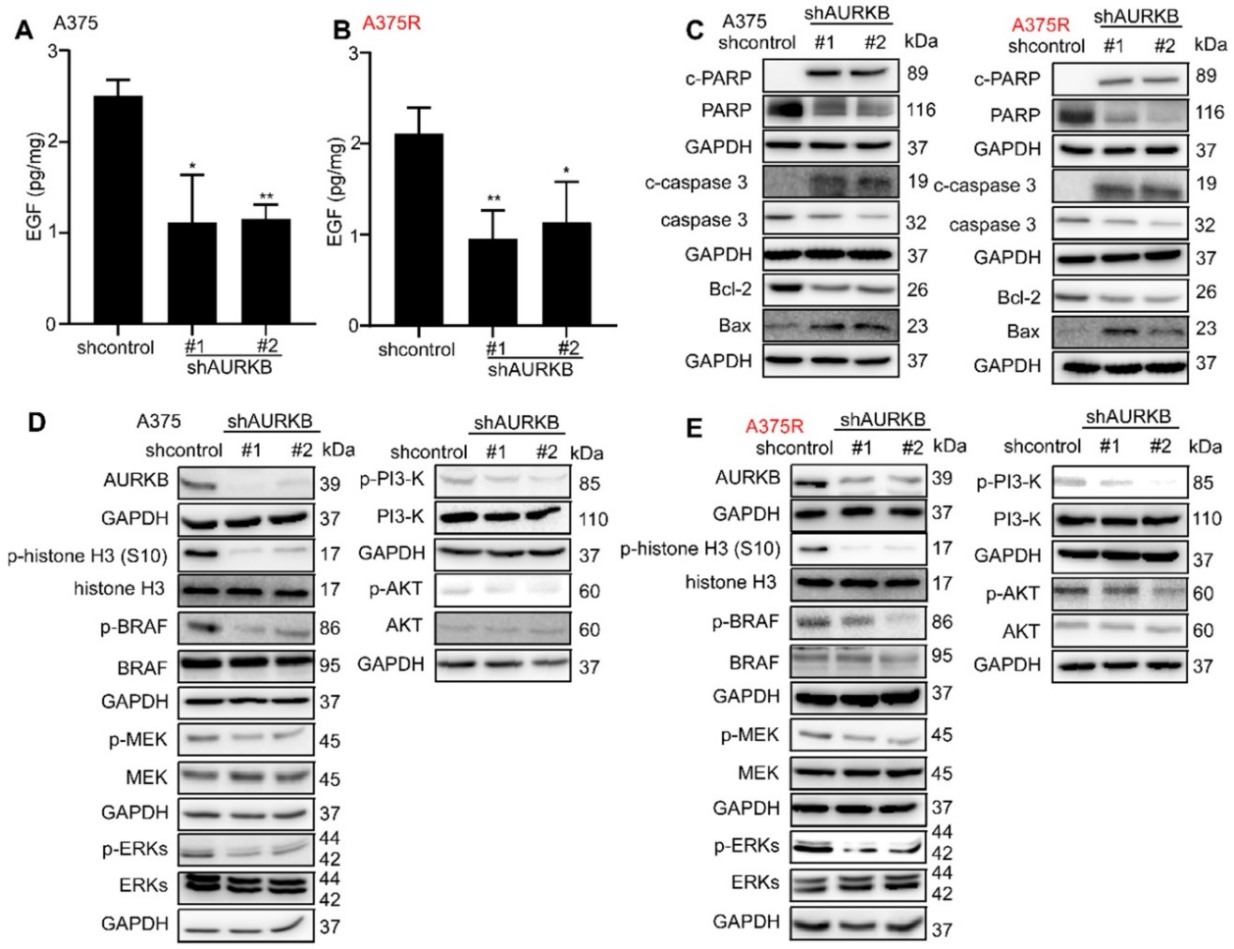
** AURKB mediates EGF associated BRAF/MEK/ERKs and PI3-K/AKT pathways. (A, B)** Knocking down of *AURKB* suppressed the level of EGF in A375 and A375R tumor from xenograft mice compared with shcontrol. The asterisks indicate a significant difference compared with the shcontrol group (*, *p* < 0.05; ** *p* < 0.01). **(C)** Knocking down of *AURKB* increased levels of c-PARP, c-caspase 3, and decreased of Bcl-2 level of A375 and A375R cell lines. **(D, E)** Western blot analysis of activation of BRAF/MEK/ERKs and PI3-K/AKT by knocking down of *AURKB* in A375 and A375R cell lines.

From the Gene Set Enrichment Analysis (GSEA) 41, 42 in GSE: 4587 (**Figure S4A, B**), we found AURKB was closely associated with the ERBB pathway. Moreover, AURKB depletion inhibits the activities of BRAF/MEK/ERKs and PI3-K/AKT pathways. The previous study showed that EGF could activate the RAS through epidermal growth factor receptor (EGFR) and activate the BRAF/MEK/ERKs pathway and PI3-K/AKT pathways 43, 44. Thus, we tested the EGF concentration from the tumors of xenograft mouse model, the results indicate that knocking down of *AURKB* could significantly reduce the EGF level in both vemurafenib-sensitive (A375, M249) and vemurafenib-resistant (A375R, M249R) xenograft tumors (**Figures 4A, B; Figures S4C, D**). Next, Western blot was utilized to detect the kinase activation related with apoptosis and the activation of BRAF/MEK/ERKs and PI3-K/AKT pathways. The cells infected with shAURKB showed pro-apoptotic signatures including increased cleaved PARP, cleaved caspase-3, Bax, and lower levels of anti-apoptotic Bcl-2 (**Figure 4C; Figure S4E**). Moreover, the Western blot results demonstrated that knocking down the expression of *AURKB* markedly suppressed activation of the BRAF/MEK/ERKs and PI3-K/AKT pathways in both vemurafenib-sensitive (A375 and M249) and vemurafenib-resistant melanoma cell lines (A375R and M249R). Meanwhile, knocking down *AURKB* decreased the phosphorylation level of histone H3 (S10), a direct substrate of AURKB (**Figures 4D, E; Figures S4F, G**). To confirm the apoptosis was mediated by inhibition of BRAF/MEK/ERKs and PI3-K/AKT pathways, the apoptosis rate was measured after knocking down *AURKB* and treated with LY294002, PI3-K inhibitor, or vemurafenib.

**Figure 5:**
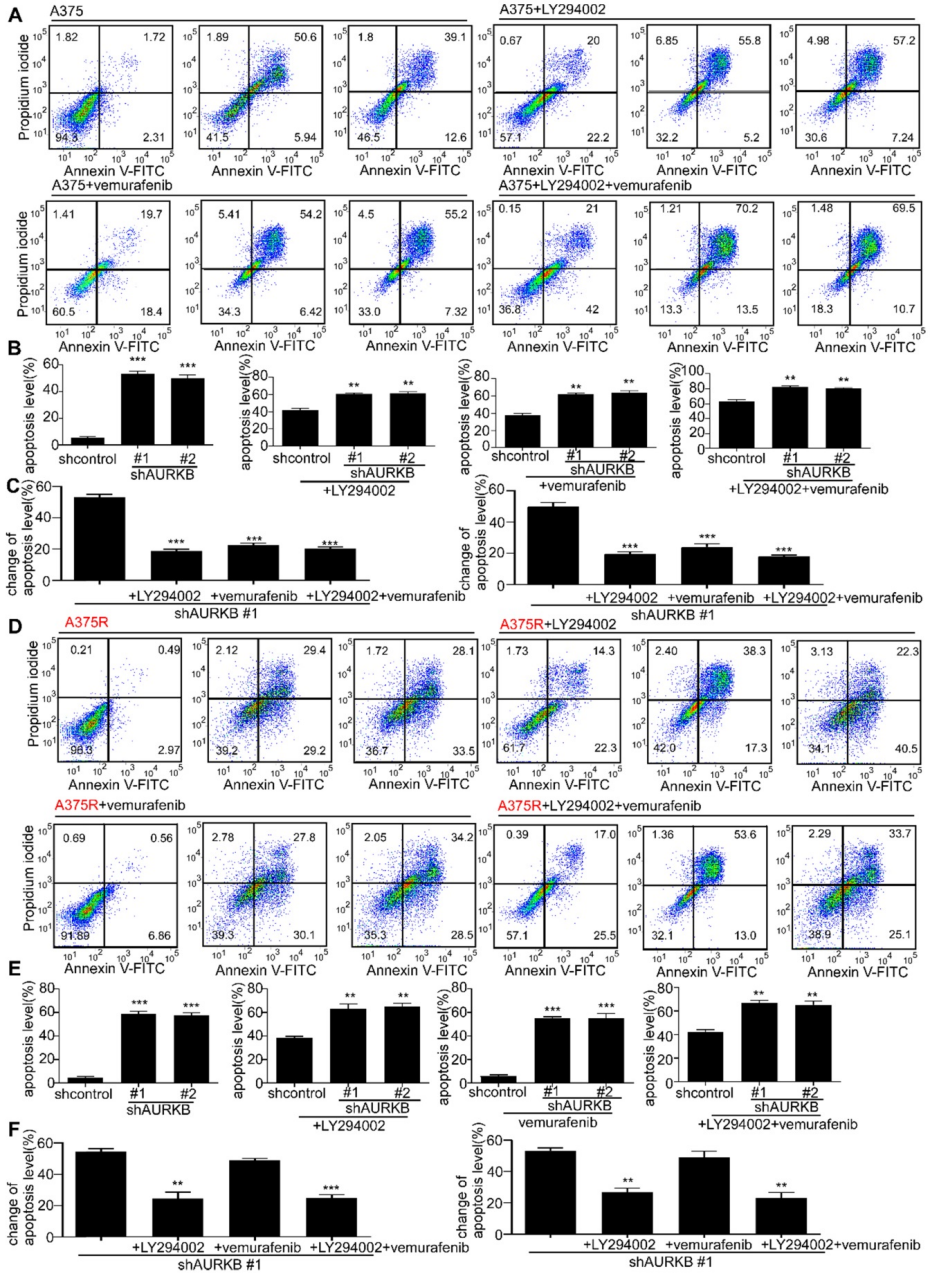
** Knocking down expression of *AURKB* would induce less additional apoptosis when BRAF/MEK/ERKs and PI3-K/AKT pathways were inhibited. (A, B)** The apoptosis rate after knocking down expression of *AURKB* with non-treatment, LY294002 treatment, vemurafenib treatment and the combination of LY294002 and vemurafenib treatment in A375 cells. **(C)** The absolute change of apoptosis rate (shAURKB - shcontrol) among A375 cells with non-treatment, LY294002 treatment, vemurafenib treatment and the combination of LY294002 and vemurafenib treatment.** (D, E)** The apoptosis rate after knocking down expression of *AURKB* with non-treatment, LY294002 treatment, vemurafenib treatment and the combination of LY294002 and vemurafenib treatment in A375R cells. **(F)** The absolute change of apoptosis rate among A375R cells with non-treatment, LY294002 treatment, vemurafenib treatment and the combination of LY294002 and vemurafenib treatment. Statistical significance was determined by one-way ANOVA and the asterisks indicate a significant change compared with the control group (**, *p* < 0.01, ***, *p* < 0.001).

The absolute change (shAURKB-shcontrol) of apoptosis was compared among the non-treatment, LY294002 treatment, vemurafenib treatment and combining LY294002 with vemurafenib treatment groups. The results showed that the additional apoptosis of shAURKB was blocked when inhibition of BRAF/MEK/ERKs or PI3-K/AKT pathways in A375 cells. Additionally, the additional apoptosis of shAURKB was blocked when inhibition of PI3-K/AKT pathway in A375R cells. These results suggested that knocking down of *AURKB*-induced apoptosis is involved in BRAF/MEK/ERKs or PI3-K/AKT pathways. (**Figures 5A-F**). Overall, knocking down the expression of *AURKB* could suppress both vemurafenib-sensitive and -resistant melanoma cell growth and induce apoptosis through the mediation of the BRAF/MEK/ERKs and PI3-K/AKT pathways.

### HI-511 binds with AURKB and BRAF V600E at the ATP binding pocket

**Figure 6:**
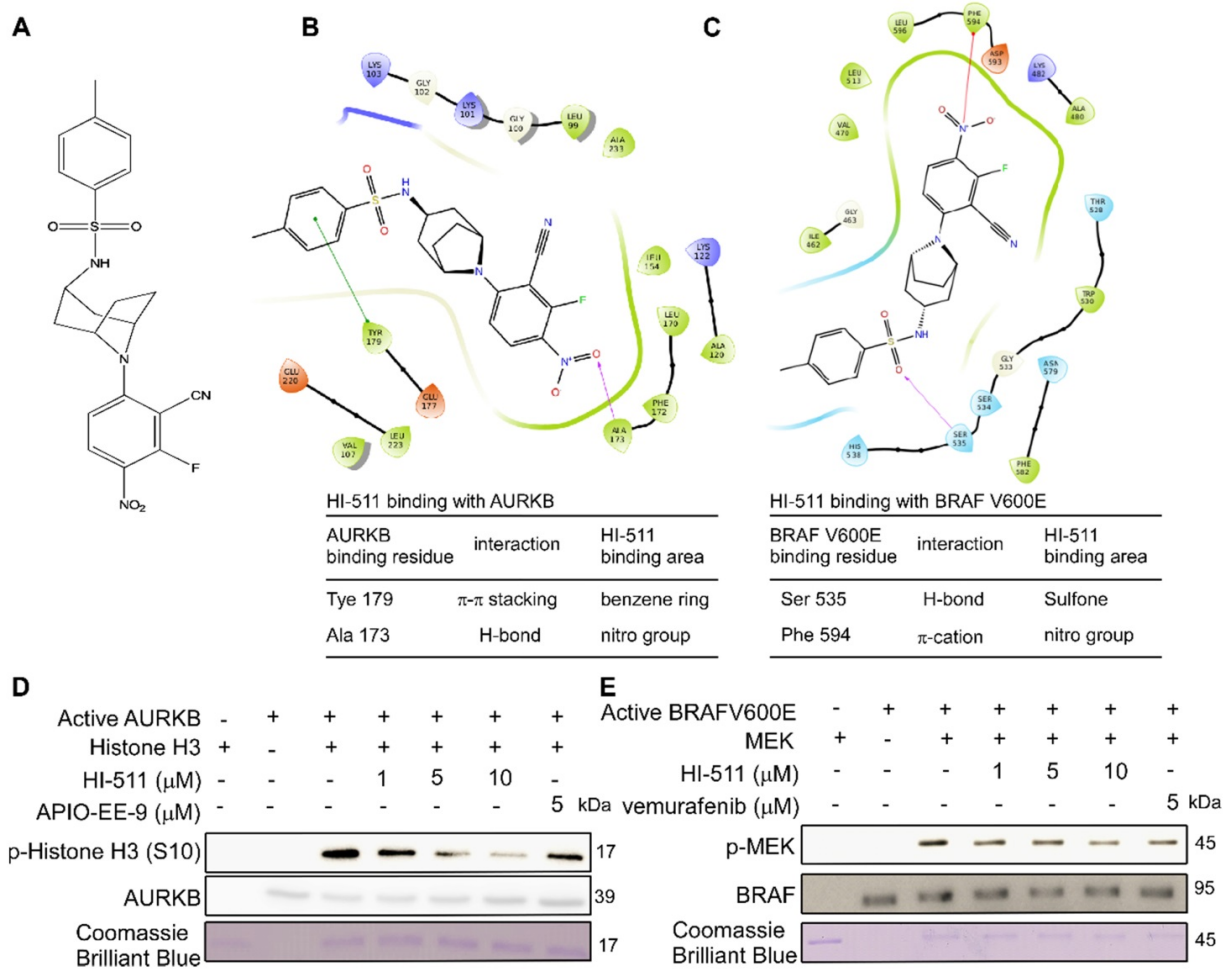
** HI-511 binds to and effectively suppresses the activation of AURKB and BRAF V600E. (A)** The structure of HI-511.** (B)** Computer docking model of HI-511 binding with AURKB. **(C)** Computer docking model of HI-511 binding with BRAF V600E. **(D)** HI-511 inhibits AURKB *in vitro*. Active kinase AURKB and HI-511 (0, 1, 5 and10 µM) or APIO-EE-9 (5 µM) were mixed with the substrate histone H3. The relative amounts of phosphorylated substrate were visualized by Western blot.** (E)** HI-511 inhibits BRAF V600E *in vitro*. Active kinase BRAF V600E and HI-511 (0, 1, 5, 10 µM) or vemurafenib (5 µM) were mixed with the substrate phosphatidylinositol. The relative amounts of phosphorylated substrate were visualized by Western blot.

A novel compound (HI-511) was synthesized (**Figure 6A**), and its structure was identified by NMR spectroscopy (**Figure S5**). Schrödinger Suite 2018 software was used to create binding models of the HI-511 docking with BRAF V600E and AURKB. The models predicted the main interaction types and binding region between HI-511 and each kinase. The phenyl ring in HI-511 binds with Tyr179 in AURKB by π-π stacking and the nitro group binds with Ala173 by a hydrogen bond. The sulfone in HI-511 binds to Ser535 on BRAF V600E by a hydrogen bond and the nitro group binds with Phe594 by π-cation interaction (**Figures 6B, C**). An *in vitro* kinase assay was conducted to determine the inhibitory effect of HI-511 on the activation of AURAB and BRAF V600E and demonstrated that HI-511 markedly inhibited both AURKB and BRAFV600E phosphorylation of their respective substrates (**Figures 6D, E**). These results indicate that HI-511 is a novel dual-target inhibitor for both AURKB and BRAF V600E.

### HI-511 mediates growth and apoptosis of vemurafenib-sensitive and -resistant melanoma cells by targeting AURKB and BRAF V600E

**Figure 7:**
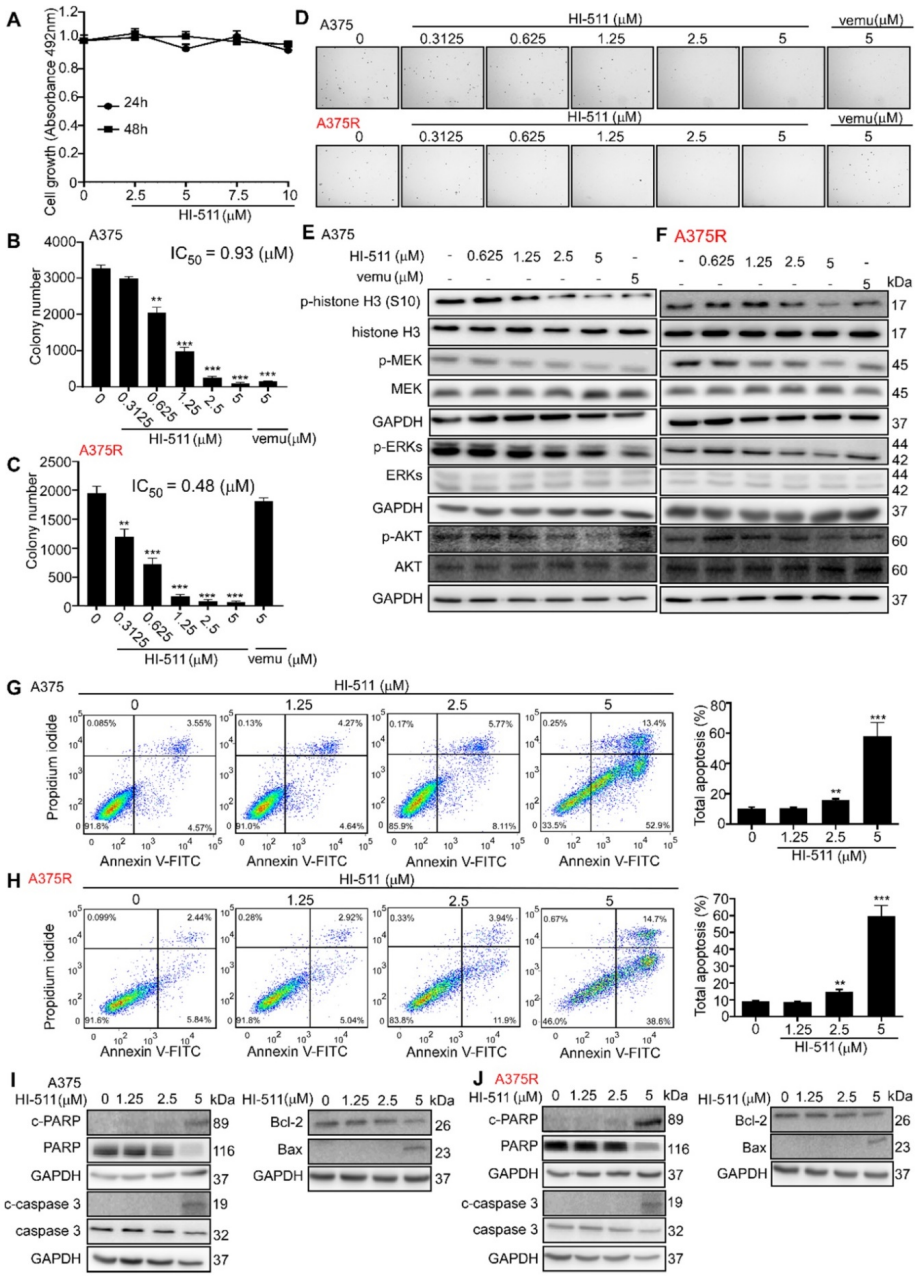
** HI-511, a dual-target inhibitor for both AURKB and BRAF V600E, affects cell growth, apoptosis, and protein expression levels in A375 and A375R melanoma cells. (A)** Melanocytes (NHEM; 1 × 10^4^/well) were seeded into 96-well plates. After incubation overnight, cells were treated with the indicated concentrations of HI-511 and incubated for 24 h or 48 h. Viability was estimated using the MTS assay as described in Materials and Methods. **(B, C)** HI-511 inhibited anchorage-independent cell growth. A375 and A375R cells (8 × 10^3^/well) were seeded into 6-well plates with 0.3% Basal Medium Eagle agar containing 10% FBS and different concentrations of HI-511 and then cultured for 2 weeks. Colonies were scored under a microscope using the Image-Pro PLUS (v6.) software program. **(D)** Representative colonies images of A375 and A375R. **(E, F)** Effects of HI-511 on the activation of histone H3, MEK, ERKs, and AKT were detected by Western blot in A375 and A375R cells. **(G, H)** A375 and A375R cells (2.5 × 10^5^/well) were incubated with HI-511 (1.25, 2.5, or 5 µM) or vehicle control for 48 h. Cells were collected and apoptosis was detected using flow cytometry and Annexin V, propidium iodide staining. **(I, J)** The cells were incubated with HI-511 or vehicle control for 48 h, then the effect of HI-511 on apoptosis-associated protein expression was determined by Western blot. Statistical significance was determined by one-way ANOVA. The asterisks indicate a significant change compared with untreated control cells (*p* < 0.01; ***, *p* < 0.001).

To determine whether HI-511 exerts any cytotoxic effects against normal melanocytes, NHEM cells were treated with different concentrations of HI-511 for 24 h or 48 h. The results showed that HI-511 was not cytotoxic at concentrations below 10 μM (**Figure 7A**). A375 or A375R cells were treated with different concentrations of HI-511 for 48 h. The results indicated HI-511 from 0.625 μM could significantly inhibit both A375 and A375R cell growth (**Figures S6A, B**). Moreover, HI-511 at 5 μM also can induce G2/M arrest in both A375 and A375R cells (**Figures S6C, D**). An anchorage-independent growth assay was conducted with different concentrations of HI-511 (0, 0.3125, 0.625, 1.25, 2.5, 5, or 10 μM) and results showed that HI-511 significantly suppressed melanoma cell growth in a dose-dependent manner. Vemurafenib treatment confirmed the sensitivity and resistance of melanoma cell lines. The IC_50_ of HI-511 for A375 and A375R was 0.93 and 0.48 μM, respectively; and the IC_50_ of HI-511 for M249 and M249R was 0.46 and 0.45 μM, respectively (**Figures 7B, C; Figures S7A, B**). Representative images of A375 and A375R colonies were shown (**Figure 7D**). Western blot analysis of the effect of HI-511 (0, 0.625, 1.25, 2.5, 5 μM) revealed a dose-dependent reduction of the phosphorylation level of histone H3 (S10), MEK, ERKs, and AKT in both vemurafenib-sensitive (A375 and M249) and -resistant melanoma cell lines (A375R and M249R; **Figures 7E, F; Figures S7C, D**). Flow cytometry results revealed that HI-511 increased apoptosis in A375, M249, A375R, and M249R cell lines in a dose-dependent manner (**Figures 7G, H; Figures S7E, F**). In addition, HI-511 treatment induced pro-apoptotic signatures including increases in cleaved PARP, cleaved caspase-3, Bax, and lower levels of anti-apoptotic Bcl-2 (**Figures 7I, J; Figures S7G, H**). Besides the BRAF V600E, HI-511 could bind with wild-type BRAF as well. The nitrile group in HI-511 binds to Cys532 on BRAF V600E by a hydrogen bond and the phenyl ring binds with Trp531 by π-π stacking. In addition, we found HI-511 inhibited wild-type BRAF activation in a dose dependent manner as well (**Figure S8A, B**). The effects of knocking down *AURKB* and HI-511 treatment on wild-type BRAF melanoma cell line (SK-MEL-31) were detected by crystal violet staining assay, the results revealed that knocking down *AURKB* and HI-511 treatment could suppress SK-MEL- 31 growth (**Figures S8C, D**). However, the inhibitory effect is less than that in BRAF V600E cells (**Figure 2A, B and Figure 7B, C**). Overall, HI-511 mediates growth and apoptosis in both vemurafenib-sensitive and -resistant melanoma cells by targeting AURKB and BRAF V600E.

### HI-511 inhibits vemurafenib-sensitive and -resistant melanoma growth in a xenograft mouse model

**Figure 8:**
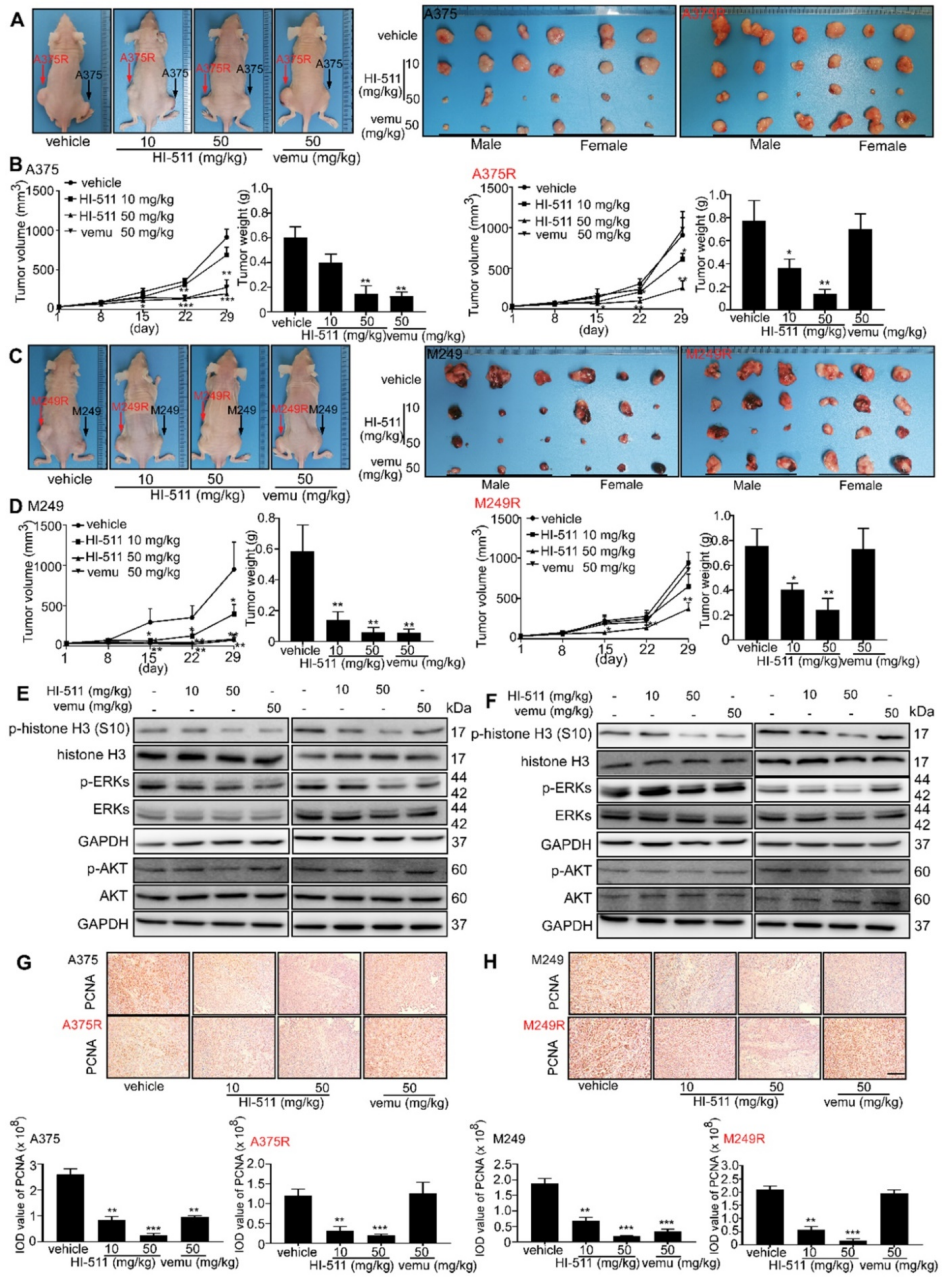
** HI-511, a dual-target inhibitor against both AURKB and BRAF V600E, inhibits vemurafenib-sensitive and -resistant melanoma in a xenograft mouse model. (A)** Tumors from A375 and A375R xenograft mouse models. **(B)** Tumor size of A375 and A375R xenografts in each group was measured every week and tumor weight was measured at day 29. **(C)** Tumors from M249 and M249R xenograft mouse models. **(D)** Tumor size of M249 and M249R xenografts in each group were measured every week and tumor weight was measured at day 29. **(E, F)** The tumors from A375, A375R, M249, or M249R cell-injected xenograft mice were used to study the effect of HI-511 on the activation of histone H3, ERKs, and AKT by Western blot. The expression of PCNA in **(G)** A375, A375R and **(H)** M249, M249R tumors in xenograft models was detected by immunohistochemistry staining; the scale bar = 100 µm. Statistical significance was determined by one-way ANOVA. The asterisks indicate a significant difference compared with the shcontrol group (*, *p* < 0.05; **, *p* < 0.01 and ***,* p* < 0.001). Vemu: vemurafenib.

A xenograft mouse experiment was conducted to determine the effect of HI-511 (0, 10, 50 mg/kg B.W.) on the growth of vemurafenib-sensitive and -resistant melanoma. HI-511 markedly attenuated tumor growth in a dose-dependent manner in both vemurafenib-sensitive and -resistant groups. Vemurafenib decreased tumor growth in the A375-inoculated cell group but failed to suppress tumor growth in the A375R-inoculated group (**Figure 8A**). Results also showed significant reductions in volume and weight of tumors from HI-511-treated mice in both vemurafenib-sensitive melanoma (A375)- and -resistant melanoma (A375R)-injected groups (**Figure 8B**). We utilized another vemurafenib-sensitive (M249) and -resistant (M249R) cell line in this xenograft mouse model. The sensitivity of M249 and resistance of M249R were confirmed and HI-511 could reduce tumor growth in a dose-dependent manner in both vemurafenib-sensitive and -resistant groups (**Figure 8C**). Tumor volume and weight were decreased in the HI-511-treated groups in both the M249 and M249R melanoma cell-injected groups and the 50 mg/kg B.W.-treated group demonstrated significant attenuation (**Figure 8D**). In addition, Western blot analysis showed that the phosphorylation levels histone H3 (S10), ERKs, and AKT in tumors was decreased in a dose-dependent manner with HI-511 treatment (**Figures 8E, F**). IHC analysis of the xenograft tumors also revealed a dose-dependent reduction in PCNA expression level (**Figures 8G, H**). The expression of c-caspase 3 and c-PARP in tumors was detected by IHC. The results showed HI-511 treatment enhanced the expression levels of c-caspase 3 and c-PARP. These results indicate that the anti-tumor mechanism of HI-511 is apoptosis of tumor cells (**Figures S9A-D**). This xenograft mouse model showed that HI-511 could suppress both vemurafenib-sensitive and -resistant melanoma growth through the inhibition of AURKB and BRAF V600E.

### The combination of HI-511 and vemurafenib treatment could inhibit vemurafenib-resistant melanoma growth

**Figure 9:**
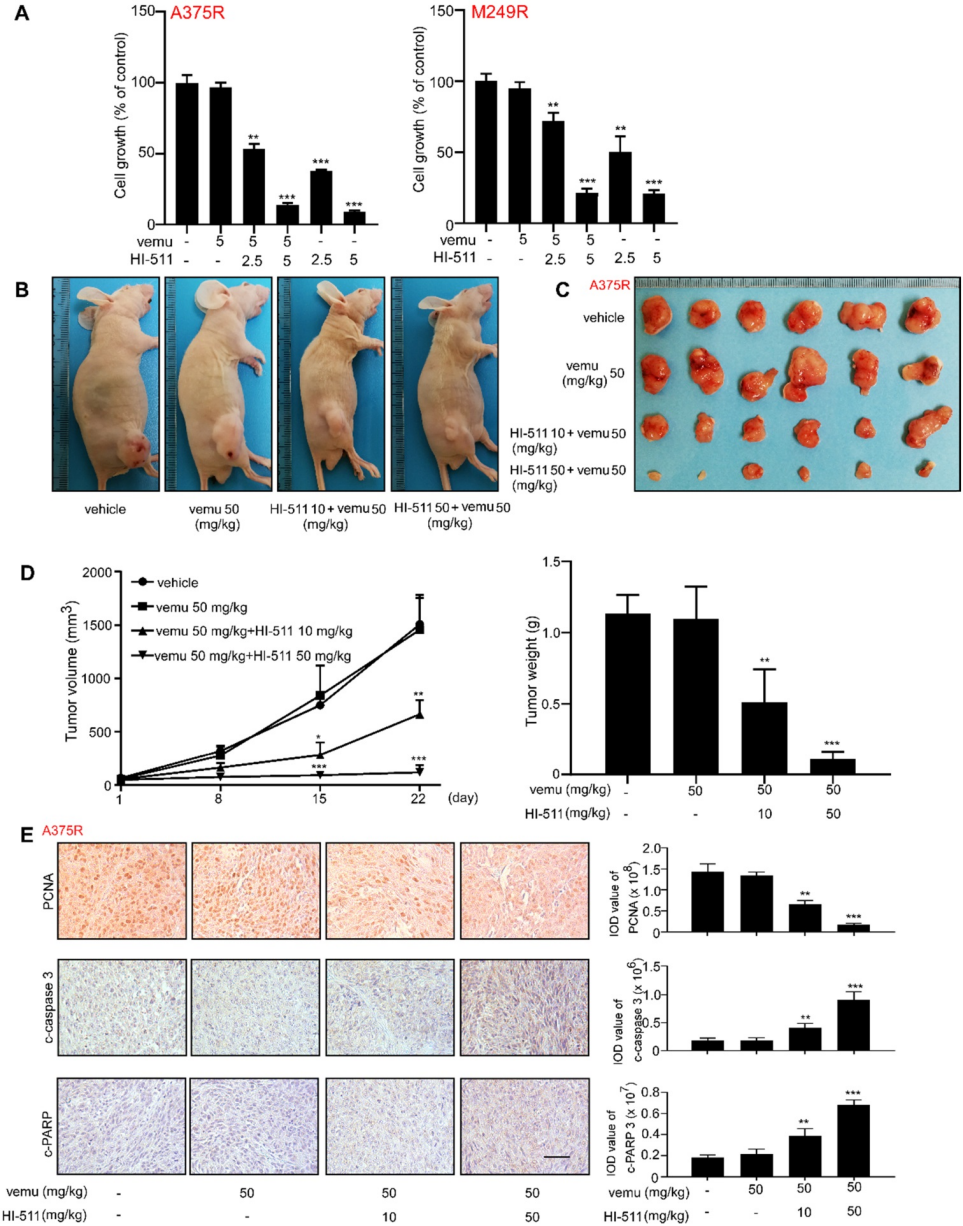
** The combination of HI-511 and vemurafenib treatment inhibits vemurafenib-resistant melanoma* in vitro* and *in vivo* (A)** vemurafenib-resistant melanoma (A375R, M249R, 2 × 10^4^/well) were seeded into 24-well plates. After incubation overnight, cells were treated with the indicated concentrations of HI-511 and vemurafenib, then incubated for 48 h. Viability was estimated using the crystal violet staining assay as described in Materials and Methods. **(B)** Representative images of nude mice from each group after 22 days drug administration. **(C)** The picture of tumors from xenograft mouse model. **(D)** Tumor size of each group was measured every week and tumor weight was measured at day 22. **(E)** The expression levels of PCNA, c-caspase 3 and c-PARP were detected by IHC; scale bars = 50 µm. Statistical significance was determined by one-way ANOVA and the asterisks indicate a significant decrease compared with vehicle control mice (*, *p* < 0.05; **, *p* < 0.01; ***, *p* < 0.001). Vemu: vemurafenib.

The effect of combination of HI-511 and vemurafenib on vemurafenib-resistant melanoma cells growth was detected by crystal violet staining assay *in vitro*, and results indicated the combination of HI-511 and vemurafenib could inhibit vemurafenib-resistant melanoma cells (A375R, M249R) growth (**Figures 9A**). A xenograft mouse experiment was conducted to determine the effect of the combination of HI-511 and vemurafenib (0, 10, 50 mg/kg HI-511 and 50 mg/kg vemurafenib B.W.) on the growth of vemurafenib-resistant melanoma. The results indicated that the combination significantly suppressed the vemurafenib-resistant melanoma tumor growth as compared to vehicle and vemurafenib treatment. Representative images of mice were shown (**Figure 9B**). Tumors from each group were shown as well (**Figure 9C**). The statistics results also showed significant reductions in volume and weight of tumors from the HI-511 and vemurafenib treatment mice (**Figure 9D**). The expression of c-caspase 3 and c-PARP in tumors was detected by IHC. The results showed that the apoptosis level was significantly increased by combined of HI-511 and vemurafenib treatment compared with vehicle group in vemurafenib-resistant melanoma (**Figure 9E**). Overall, the combination of HI-511 and vemurafenib treatment could suppress vemurafenib-resistant melanoma growth both *in vitro* and *in vivo*.

### HI-511 suppresses tumor growth in the *BRAF V600E* mutant and the *PTEN-* loss mouse models

**Figure 10:**
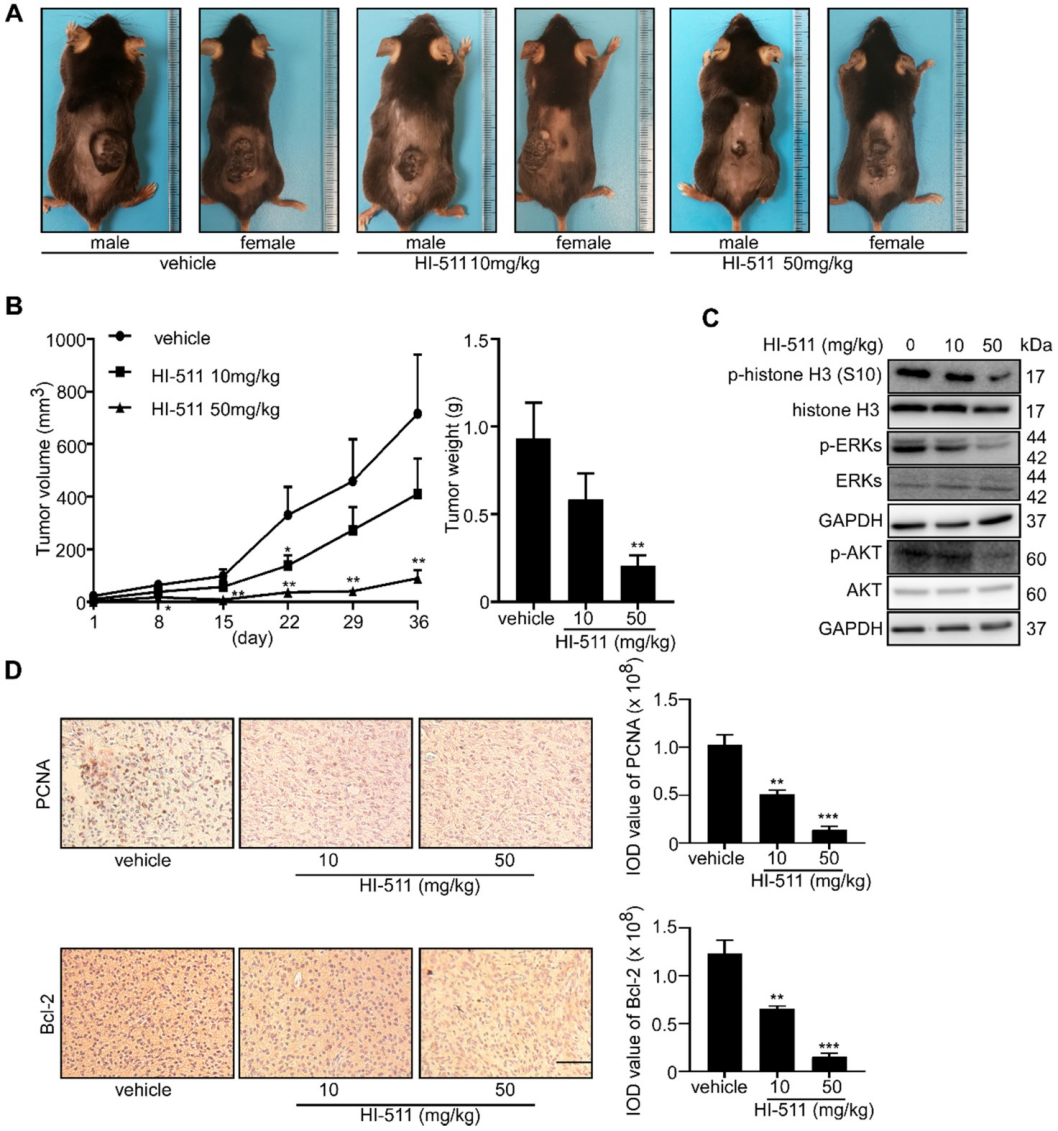
** HI-511, a dual-target inhibitor against both AURKB and BRAF V600E, inhibited melanoma development in *BRAFV 600E*/*PTEN*-null mice.** Melanoma was initiated in *BRAF V600E*/*PTEN*-null mice (6-8 weeks old) by local administration of 2.5 µL of 5 mM 4-HT by local application to the dorsal skin. At 23 days later, when the animals had readily measurable melanoma lesions, mice were randomly divided into 3 groups that were administered HI-511 (10 mg/kg B.W. n = 8), HI-511 (50 mg/kg B.W. n = 8) or vehicle control (n = 8). **(A)** Representative images of vehicle control or HI-511-treated mice after 36 days drug administration. **(B)** Tumor size in each of the treatment groups was measured every week. Tumor weight was measured at day 36. **(C)** Mice were euthanized, and melanoma specimens were disrupted and the expression of p-histone H3, histone 3, p-ERKs, ERKs, p-AKT, AKT, and GAPDH was assessed by Western blot. **(D)** Melanoma specimens were prepared for IHC staining for PCNA and Bcl-2; scale bars = 100 µm. Statistical significance was determined by one-way ANOVA and the asterisks indicate a significant decrease compared with vehicle control mice (*, *p* < 0.05; **, *p* < 0.01; ***, *p* < 0.001). Vemu: vemurafenib.

The *BRAF V600E* mutant/*PTEN*-loss mouse experiment was conducted to determine the effect of HI-511 on melanoma development in mice. Treatment with HI-511 (10, 50 mg/kg B.W.) strongly inhibited tumor growth in a dose-dependent manner compared with the vehicle control-treated group (**Figure 10A**). In addition, HI-511 significantly reduced tumor volume and weight at a dose of 50 mg/kg per day (**Figure 10B**). In addition, Western blot analysis showed dose-dependent decreased phosphorylation of histone H3 (S10), ERKs, and AKT in tumors from mice treated with HI-511 (**Figure 10C**). IHC analysis of tumors revealed a dose-dependent reduction in PCNA and Bcl-2 expression levels (**Figure 10D**). This animal model validated the therapeutic effect of HI-511 on melanoma development in mice.

## Discussion

Chemical therapeutic strategies against melanoma are widely used in clinical therapy 45-47. One of the most serious problems in treatment is the development of drug resistance 12, 48. In this study, we identified AURKB as a critical target for drug-sensitive and - resistance melanoma treatment and found that HI-511 effectively suppressed development of vemurafenib-resistant melanoma by targeting both AURKB and BRAF V600E.

BRAF V600E is found in about 50% of melanoma patients 49 and a chemical therapeutic strategy has been approved by FDA. However, the overall survival rate is still unsatisfactory and the cause of this is drug resistance. Development of novel targets for melanoma treatment is a potential strategy by which to overcome drug resistance. Analysis of databases revealed the important role of AURKB in melanoma development. AURKB is overexpressed in melanoma, and the overexpression is associated with a reduced probability of survival of melanoma patients (**Figure 1**). AURKB is considered to be a critical kinase related to cell growth and apoptosis 50, and the safety of AURKB as a therapeutic target has been confirmed 47, 51. Previous reports indicated that AURKB expression is regulated by the MEK/ERKs pathway 52, 53. In approximately 80% of patients with resistant melanoma, the MAPK pathway is reactivated 13. In this study, we comprehensively studied the role and function of AURKB in melanoma, especially the interaction between AURKB and the BRAF/MEK/ERKs and PI3-K/AKT pathways. Our results indicated that AURKB could mediate both of these pathways. Consequently, partially silencing AURKB induced apoptosis in both vemurafenib-sensitive and -resistant melanoma cell lines equally (**Figures 2C-F**). These results indicate that AURKB could be a potential target for both drug-sensitive and -resistant melanoma therapy.

A combination of multiple targets could help to extend the median progression-free survival of melanoma patients. The FDA has approved mutant BRAF inhibitors for melanoma therapy. From the results of randomized clinical trials, the patients with a BRAF mutation experienced a longer median progression-free survival from a combination of a mutant BRAF inhibitor and a MEK inhibitor (vemurafenib-cobimetinib group = 9.9 months vs : vemurafenib-placebo group = 6.2 months, *p* < 0.001; and Dabrafenib-trametinib group = 9.4 months vs dabrafenib only = 5.8 months, *p* < 0.001) 15, 17. Our results demonstrated that AURKB could be a potential target for melanoma treatment. Previous studies showed that an AURKB inhibitor could help overcome drug resistance in cancer. AURKB was reported as a potential target in non-small cell lung cancer (NSCLC) with anti-EGFR resistance 54 and AURKB inhibitors reportedly could also help to overcome cetuximab resistance in head and neck squamous cell carcinoma (HNSCC) 30. Therefore, a dual-target inhibitor for both AURKB and BRAF V600E might be an effective approach for overcoming drug-resistant melanoma.

Computer simulation and a computational model generated by Schrödinger software provided a powerful binding model between the compound and protein base involving non-covalent bonds, including hydrogen bonding 34, 55. Using this system and an *in vitro* kinase assay, we found that HI-511 is a dual-target inhibitor against both AURKB and BRAF V600E** (Figure 6)**. HI-511 mediated growth arrest of melanoma cells and apoptosis in 2 panels of melanoma cells. Interestingly, HI-511 showed a higher inhibitory effect in vemurafenib-resistant melanoma compared with vemurafenib-sensitive melanoma (IC_50_ of A375 = 0.93 μM and IC_50_ of A375R = 0.48 μM; **Figure 7B, C**). In a xenograft model, two flank inoculations provided the same environment for tumor growth. This design could exclude other factors that might affect tumor growth, and only exhibit growth tendency of different cell lines. Importantly, HI-511 strongly inhibited both vemurafenib-sensitive and -resistant tumor growth whereas vemurafenib alone showed an inhibitory effect against only drug-sensitive tumor growth **(Figure 8)**. In addition, HI-511 significantly attenuated tumor growth in the *BRAF V600E* mutant and *PTEN*-loss mouse model, which mimics human tumor growth 56. Our results indicate that HI-511 should be an effective drug against melanoma and drug-resistant melanoma treatment.

In the current study, total survival rate is inversely associated with *AURKB* expression level. Patients with high *AURKB* expression level may have poor prognosis comparing with patients with low *AURKB* expression level (**Figure 1E**). Additionally, the previous studies also showed AURKB might be a proliferation-independent prognostic factor in breast and renal cancers. In breast cancer, the level of *AURKB* is overexpressed and related to low survival rate of patient. In rental cancer, the expression of *AURKB* associated with clinical and pathological characteristics of patients with rental cancer and its expression levels were independent prognostic factors for rental cancer 57, 58. These evidences suggested that AURKB could be not only a potential target but also be a proliferation-independent prognostic factor.

In summary, we showed AURKB to be a new target for both drug-sensitive and -resistant melanoma treatment. We identified a unique role for AURKB in melanoma, through the mediation of both the BRAF/MEK/ERKs and PI3-K/AKT pathways. Notably, we developed a dual-target inhibitor against both AURKB and BRAF V600E, which suppresses both drug-sensitive and -resistant melanoma development. These findings could provide a novel option with which to overcome drug-resistant melanoma and provide hope for clinical use.

## Supplementary Material

Supplementary figures.

## Abbreviations

AURKB: aurora kinase B
PTEN: phosphatase and tensin homolog
MEK: mitogen-activated protein kinase kinase
ERKs: extracellular signal-regulated kinases
PI3-K: phosphoinositide 3-kinase
AKT: protein kinase B
NMR: nuclear magnetic resonance
SDS: sodium dodecyl sulfate
PARP: poly (ADP-ribose) polymerase
Bcl-2: B-cell lymphoma 2
Bax: Bcl-2-associated X protein
GAPDH: glyceraldehyde 3-phosphate dehydrogenase
DMEM: Dulbecco's Modified Eagle Medium
FBS: fetal bovine serum
PCNA: proliferating cell nuclear antigen
vemu: vemurafenib
BIRC5: baculoviral inhibitor of apoptosis repeat-containing 5
BUB1: budding uninhibited by benzimidazoles 1
CDCA5: cell division cycle associated 5
CENPN: centromere protein N
NDC80: NDC80 kinetochore complex component
NUF2: NUF2 component of NDC80 kinetochore complex
