# Supplementary material for: HI-511 overcomes melanoma drug resistance via targeting AURKB and BRAF V600E
Source: Theranostics. 2020 Aug 1;10(21):9721–40. doi: 10.7150/thno.44342 (PMC7449901; doi:10.7150/thno.44342)
Supplement: Supplementary file 1 — Supplementary figures. [file thnov10p9721s1.pdf]

Supplementary Figure legends

Figure S1. **The overall survival rates of melanoma patients with different expression levels of genes listed in a Venn diagram in Figure 1D**. **(A)** The overall survival rates of melanoma patients with different *BIRC5* expression were not significantly different (p-value = 0.08). **(B)** The overall survival rates of melanoma patients with different *BUB1* expression were not significantly different (p-value = 0.91). **(C)** The overall survival rates of melanoma patients with different *CDCA5* expression were not significantly different (p-value = 0.24). **(D)** The overall survival rates of melanoma patients with different *CENPN* expression were not significantly different (p-value = 0.64). **(E)** The overall survival rates of melanoma patients with different *NDC80* expression were not significantly different (p-value = 0.62). **(F)** The overall survival rates of melanoma patients with different *NUF2* expression were not significantly different (p-value = 0.54).

Figure S2. **Knocking down expression of *AURKB* mediates melanoma cell cycle.** Knocking down expression of *AURKB* of **(A)** A375 and **(B)** A375R was established. The cells (2.5×10^5/well) were seeded into 60-mm dishes. Samples were analyzed by using a FACSCalibur flow cytometer.

Figure S3. Knocking down of *AURKB* mediates expression of c-caspase 3 and c-PARP in xenograft melanoma specimens. Melanoma specimens from **(A)** A375, **(B)** A375R, **(C)** M249 and **(D)** M249R xenograft models were prepared for c-caspase

3 and c-PARP analysis by IHC staining; scale bars = 100 μm. Statistical significance was determined by one-way ANOVA and the asterisks indicate a significant decrease compared with vehicle control mice (***, $p < 0.001$).

Figure S4. Knocking down expression of *AURKB* mediates EGF associated BRAF/MEK/ERKs and PI3-K/AKT pathways in melanoma. (A) The GSEA enrichment plot of the ERBB signaling pathway is associated with expression of *AURKB*; the expression data was from GSE: 4587. **(B)** Normalized expression profile with different *AURKB* expressing samples. Heat map was generated by GSEA. **(C, D)** Knocking down expression of *AURKB* decreased EGF level by ELISA kit in M249 and M249R. **(E)** Knocking down expression of *AURKB* increases in levels of cleaved PARP, cleaved caspase 3, Bax and decreases in Bcl-2 by Western blot. **(F, G)** Knocking down expression of *AURKB* mediates activation of histone H3, BRAF/MEK/ERKs and PI3-K/AKT in M249 and M249R cell lines. Statistical significance was determined by one-way ANOVA and the asterisks indicate a significant change compared with the control group (*, $p < 0.05$; **, $p < 0.01$).

Figure S5. **HI-511 synthesis.** The synthesis of HI-511 and NMR spectrum of the products.

Figure S6. HI-511 inhibits cell growth and mediates cell cycle in both A375 and A375R cells. (A) A375 **(B)** A375R (8×10^3/well) cells were seeded into 96-well

plates. After incubation overnight, cells were treated with the indicated concentrations of HI-511 and incubated for 48 h. Viability was estimated using the MTS assay as described in Materials and Methods. **(C)** A375 and **(D)** A375R cells were seeded (2.5 × 10^5/well) into 60-mm dishes. Treatment with DMSO or 5 μM HI-511 for 48 h. Samples were analyzed by using a FACSCalibur flow cytometer. Statistical significance was determined by one-way ANOVA and the asterisks indicate a significant change compared with the control group (*, $p < 0.05$; **, $p < 0.01$; ***, $p < 0.001$).

Figure S7. **HI-511, a dual-target inhibitor against both AURKB and BRAF V600E, affects growth, apoptosis, and protein expression levels in M249 and M249R melanoma cells. (A, B)** HI-511 inhibits anchorage-independent cell growth. Cells (8 × 10^3/well) were seeded into 6-well plates with 0.3% Basal Medium Eagle agar containing 10% FBS and different concentrations of HI-511 and then cultured for 2 weeks. Colonies were scored under a microscope by using the Image-Pro PLUS (v6.) software program. Effect of HI-511 and the activation of histone H3, MEK, ERKs, and AKT was detected by Western blot in **(C)** M249 and **(D)** M249R cells. **(E, F)** M249 and M249R cells (2.5 × 10^5/well) were incubated with HI-511 (1.25, 2.5, or 5 μM) or vehicle control for 48 h. Cells were collected and apoptosis was detected by flow cytometry with Annexin V and propidium iodide staining. **(G, H)** The cells were incubated with HI-511 or vehicle control for 48 h, then the effect of HI-511 on apoptosis-associated protein expression was determined by Western blot. Statistical

significance was determined by one-way ANOVA. The asterisks indicate a significant change compared with untreated control cells (*, $p < 0.05$; **, $p < 0.01$; ***, $p < 0.001$). Vemu: vemurafenib.

Figure S8. HI-511 binds to and suppresses the activation of wild type BRAF. (A) Computer docking model of HI-511 binding with wild type BRAF. **(B)** HI-511 inhibits wild type BRAF *in vitro*. Active kinase wild type BRAF and HI-511 (0, 1, 5, 10 μM) or vemurafenib (5 μM) were mixed with the substrate phosphatidylinositol. **(C)** Knocking down of *AURKB* inhibits BRAF wild type melanoma cell (SK-MEL-31) growth. **(D)** HI-511 suppresses BRAF wild type melanoma cell (SK-MEL-31) growth. Statistical significance was determined by one-way ANOVA. The asterisks indicate a significant change compared with untreated control cells (**, $p < 0.01$; ***, $p < 0.001$).

Figure S9. **HI-511 mediates the expression of apoptosis markers c-caspase 3 and c-PARP.** Melanoma specimens from **(A)** A375, **(B)** A375R, **(C)** M249 and **(D)** M249R xenograft models were prepared for IHC staining for c-caspase 3 and c-PARP; scale bars = 100 μm. Statistical significance was determined by one-way ANOVA and the asterisks indicate a significant decrease compared with vehicle control mice (*, $p < 0.05$; **, $p < 0.01$; ***, $p < 0.001$). Vemu: vemurafenib.

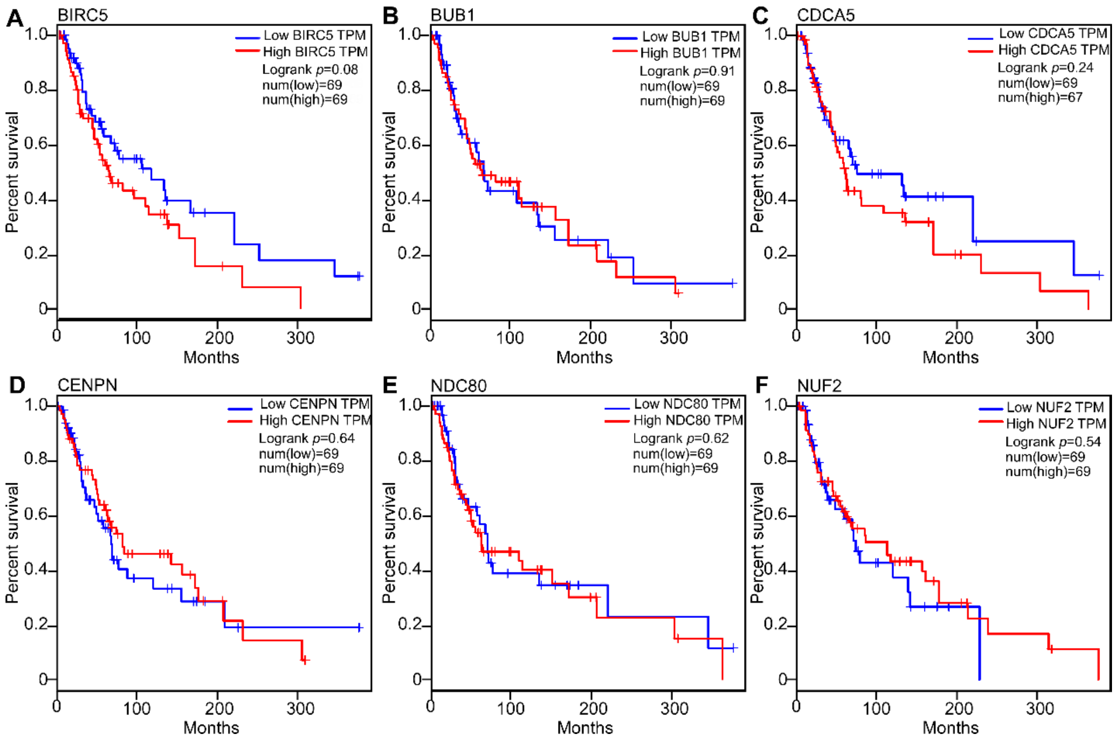
A
BIRC5
Low BIRC5 TPM
High BIRC5 TPM
Logrank p=0.08
num(low)=69
num(high)=69
B
BUB1
Low BUB1 TPM
High BUB1 TPM
Logrank p=0.91
num(low)=69
num(high)=69
C
CDCA5
Low CDCA5 TPM
High CDCA5 TPM
Logrank p=0.24
num(low)=69
num(high)=67
D
CENPN
Low CENPN TPM
High CENPN TPM
Logrank p=0.64
num(low)=69
num(high)=69
E
NDC80
Low NDC80 TPM
High NDC80 TPM
Logrank p=0.62
num(low)=69
num(high)=69
F
NUF2
Low NUF2 TPM
High NUF2 TPM
Logrank p=0.54
num(low)=69
num(high)=69
Percent survival
Months
1.0
0.8
0.6
0.4
0.2
0
100
200
300

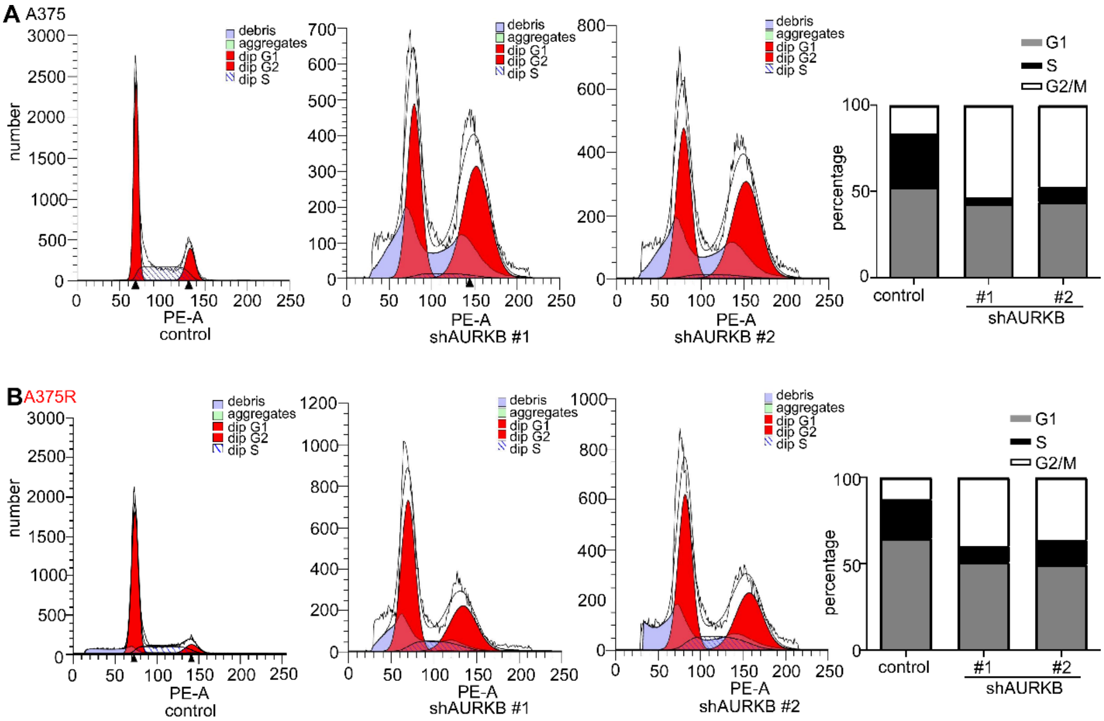
A A375
debris
aggregates
dip G1
dip G2
dip S
number
PE-A
control
shAURKB #1
shAURKB #2
G1
S
G2/M
percentage
control
#1
#2
shAURKB
B A375R
debris
aggregates
dip G1
dip G2
dip S
number
PE-A
control
shAURKB #1
shAURKB #2
G1
S
G2/M
percentage
control
#1
#2
shAURKB

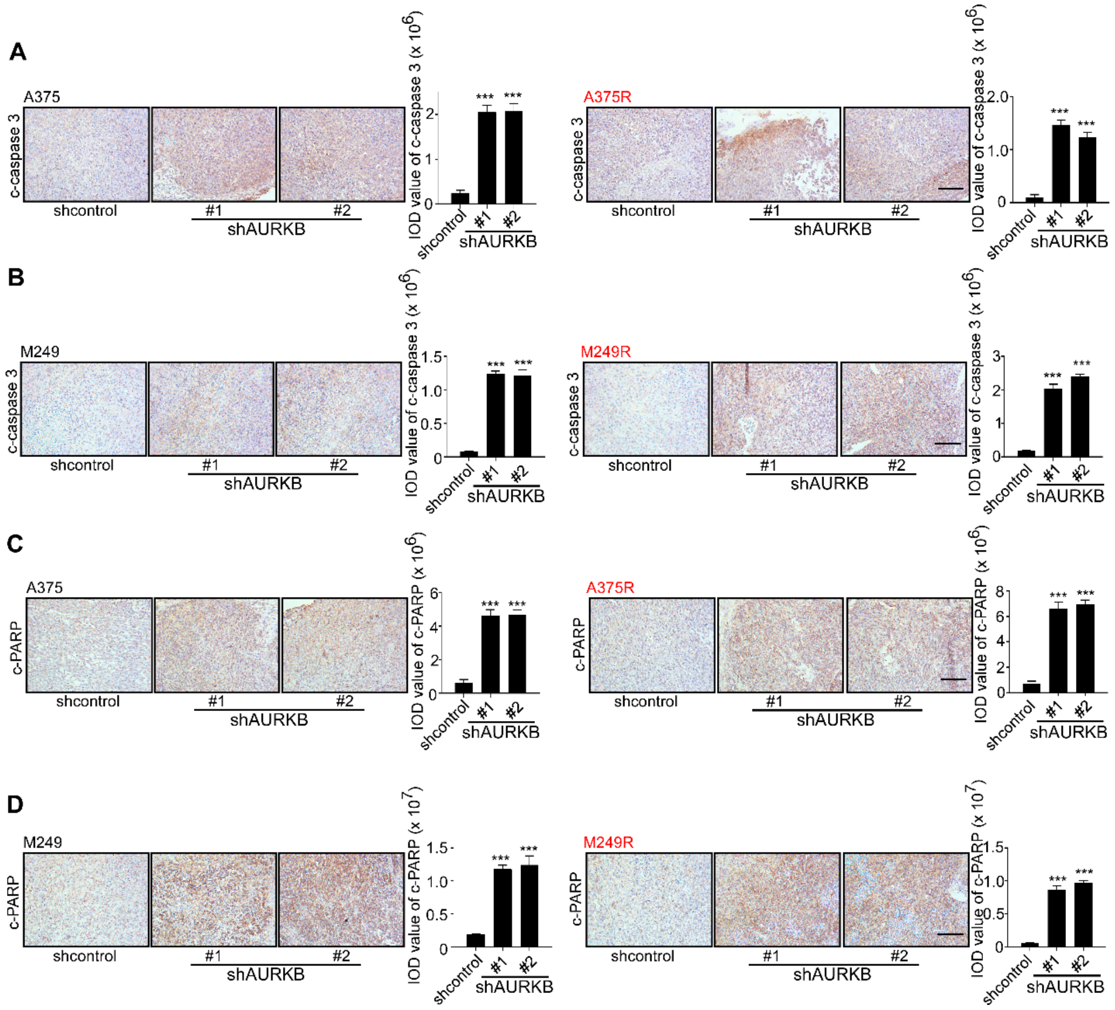
A
A375
c-caspase 3
shcontrol
#1
#2
shAURKB
IOD value of c-caspase 3 (x 10^6)
A375R
B
M249
M249R
C
c-PARP
IOD value of c-PARP (x 10^6)
D
IOD value of c-PARP (x 10^7)
***

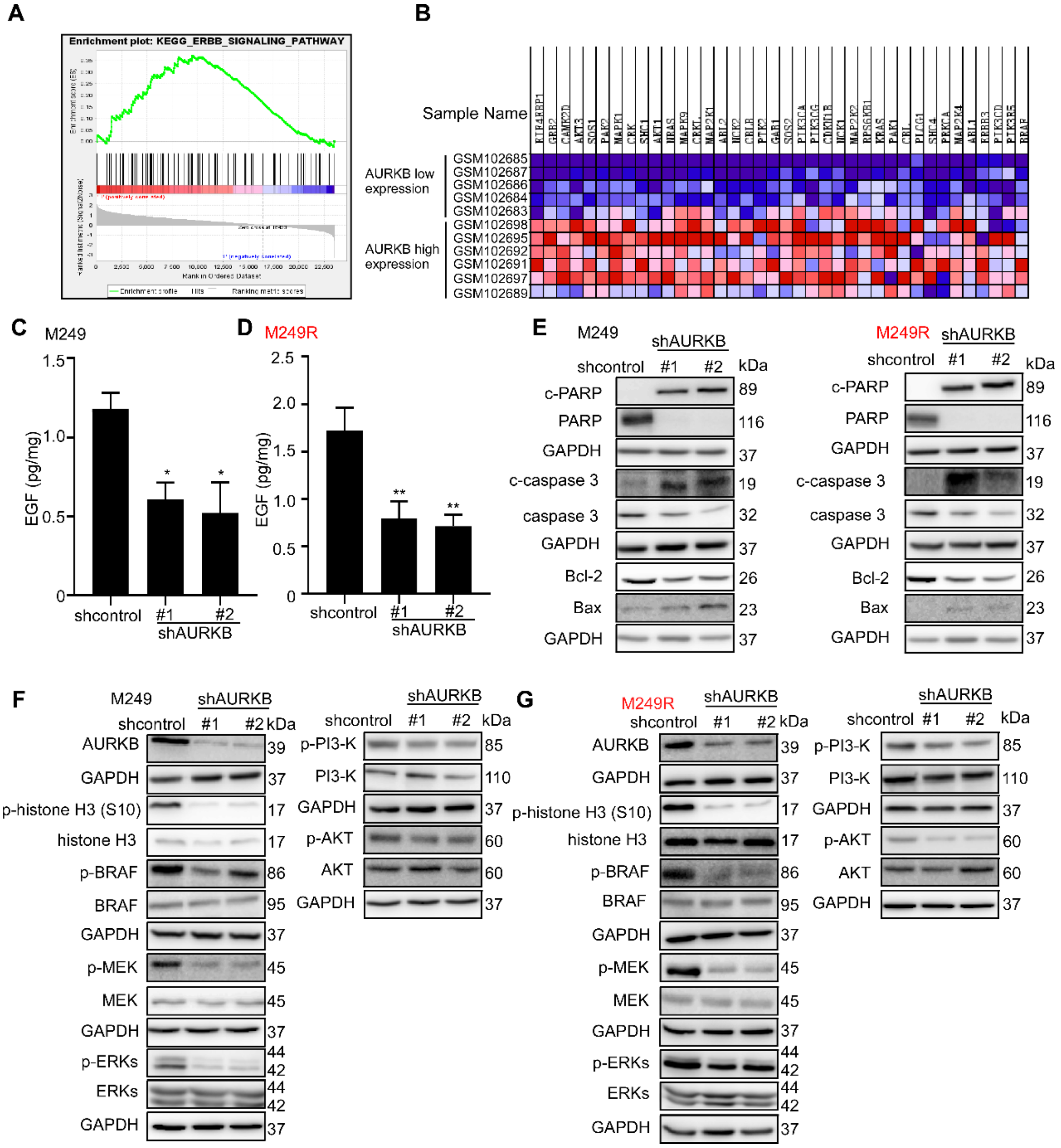
A
Enrichment plot: KEGG_ERBB_SIGNALING_PATHWAY
Rank in Ordered Dataset
Enrichment profile
Hits
Ranking metric scores
B
Sample Name
EIF4EBP1 GRB2 CAMK2D AKT3 SOS1 PAK2 MAPK1 CRK SHC1 AKT1 NRAS MAPK9 CRKL MAP2K1 ABL2 NCK2 CBLB PTK2 GAB1 SOS2 PIK3CA PIK3CG CDKN1B NCK1 MAP2K2 RPS6KB1 KRAS PAK1 CBL PLCG1 SHC4 PRKCA MAP2K4 ABL1 ERBB3 PIK3CD PIK3R5 BRAF
AURKB low expression
AURKB high expression
GSM102685
GSM102687
GSM102686
GSM102684
GSM102683
GSM102698
GSM102695
GSM102692
GSM102691
GSM102697
GSM102689
C
M249
EGF (pg/mg)
shcontrol
#1
#2
shAURKB
D
M249R
EGF (pg/mg)
shcontrol
#1
#2
shAURKB
E
M249
shAURKB
shcontrol #1 #2 kDa
c-PARP 89
PARP 116
GAPDH 37
c-caspase 3 19
caspase 3 32
GAPDH 37
Bcl-2 26
Bax 23
GAPDH 37
M249R
shAURKB
shcontrol #1 #2 kDa
c-PARP 89
PARP 116
GAPDH 37
c-caspase 3 19
caspase 3 32
GAPDH 37
Bcl-2 26
Bax 23
GAPDH 37
F
M249
shAURKB
shcontrol #1 #2 kDa
AURKB 39
GAPDH 37
p-histone H3 (S10) 17
histone H3 17
p-BRAF 86
BRAF 95
GAPDH 37
p-MEK 45
MEK 45
GAPDH 37
p-ERKs 44 42
ERKs 44 42
GAPDH 37
shAURKB
shcontrol #1 #2 kDa
p-PI3-K 85
PI3-K 110
GAPDH 37
p-AKT 60
AKT 60
GAPDH 37
G
M249R
shAURKB
shcontrol #1 #2 kDa
AURKB 39
GAPDH 37
p-histone H3 (S10) 17
histone H3 17
p-BRAF 86
BRAF 95
GAPDH 37
p-MEK 45
MEK 45
GAPDH 37
p-ERKs 44 42
ERKs 44 42
GAPDH 37
shAURKB
shcontrol #1 #2 kDa
p-PI3-K 85
PI3-K 110
GAPDH 37
p-AKT 60
AKT 60
GAPDH 37

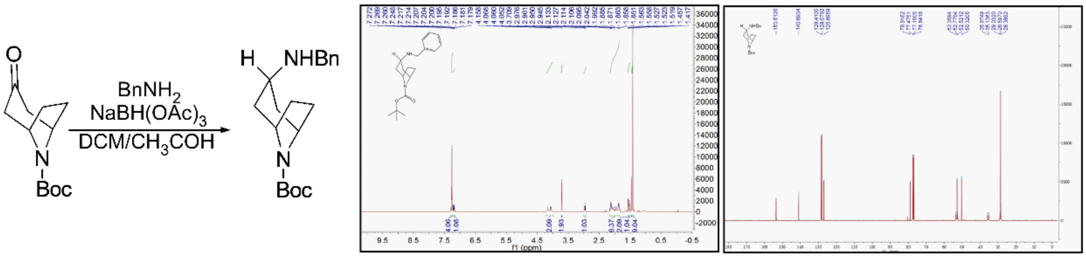

O
BnNH2
NaBH(OAc)3
DCM/CH3COH
N
Boc
H
NHBn

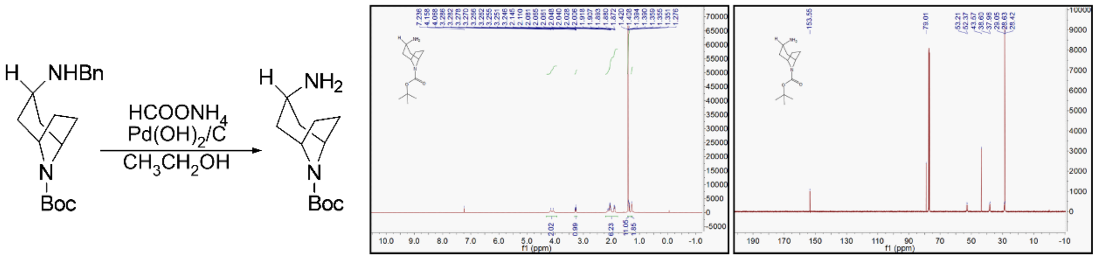

H
NHBn
N
Boc
HCOONH4
Pd(OH)2/C
CH3CH2OH
NH2

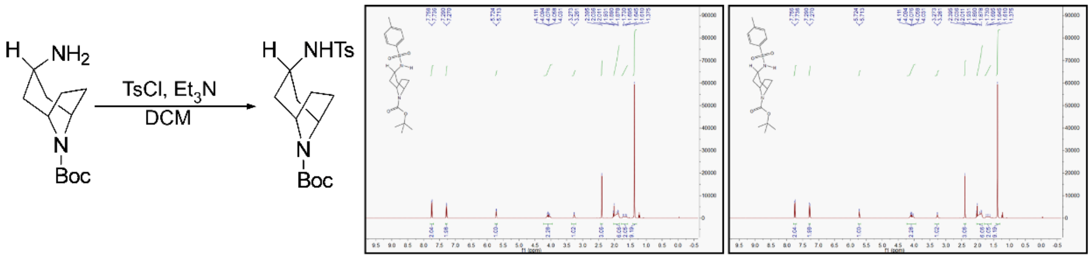

H
NH2
N
Boc
TsCl, Et3N
DCM
NHTs

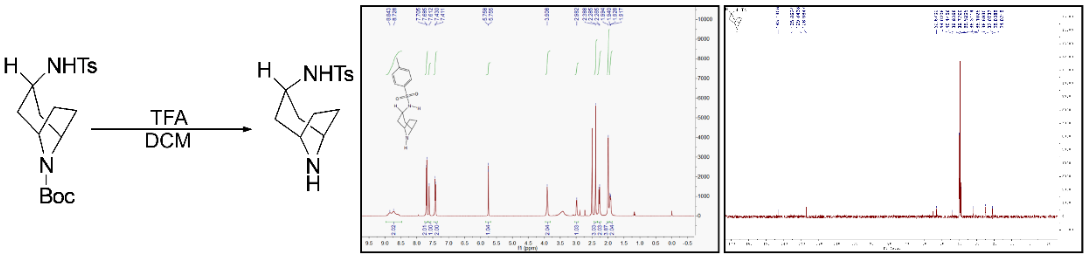

H
NHTs
N
Boc
TFA
DCM
NH

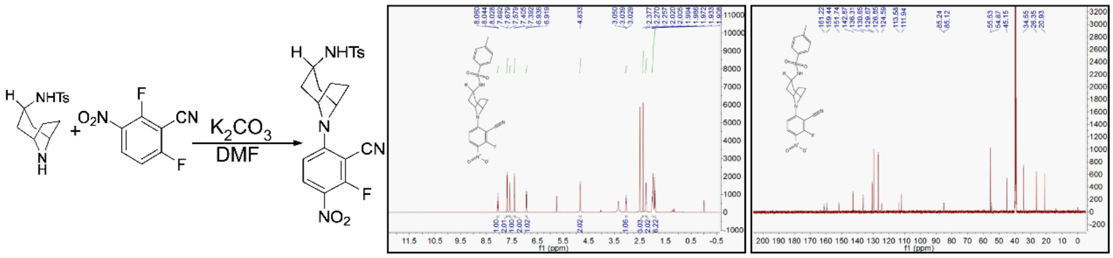

H
NHTs
N
H
+
O2N
F
CN
F
K2CO3
DMF
NO2

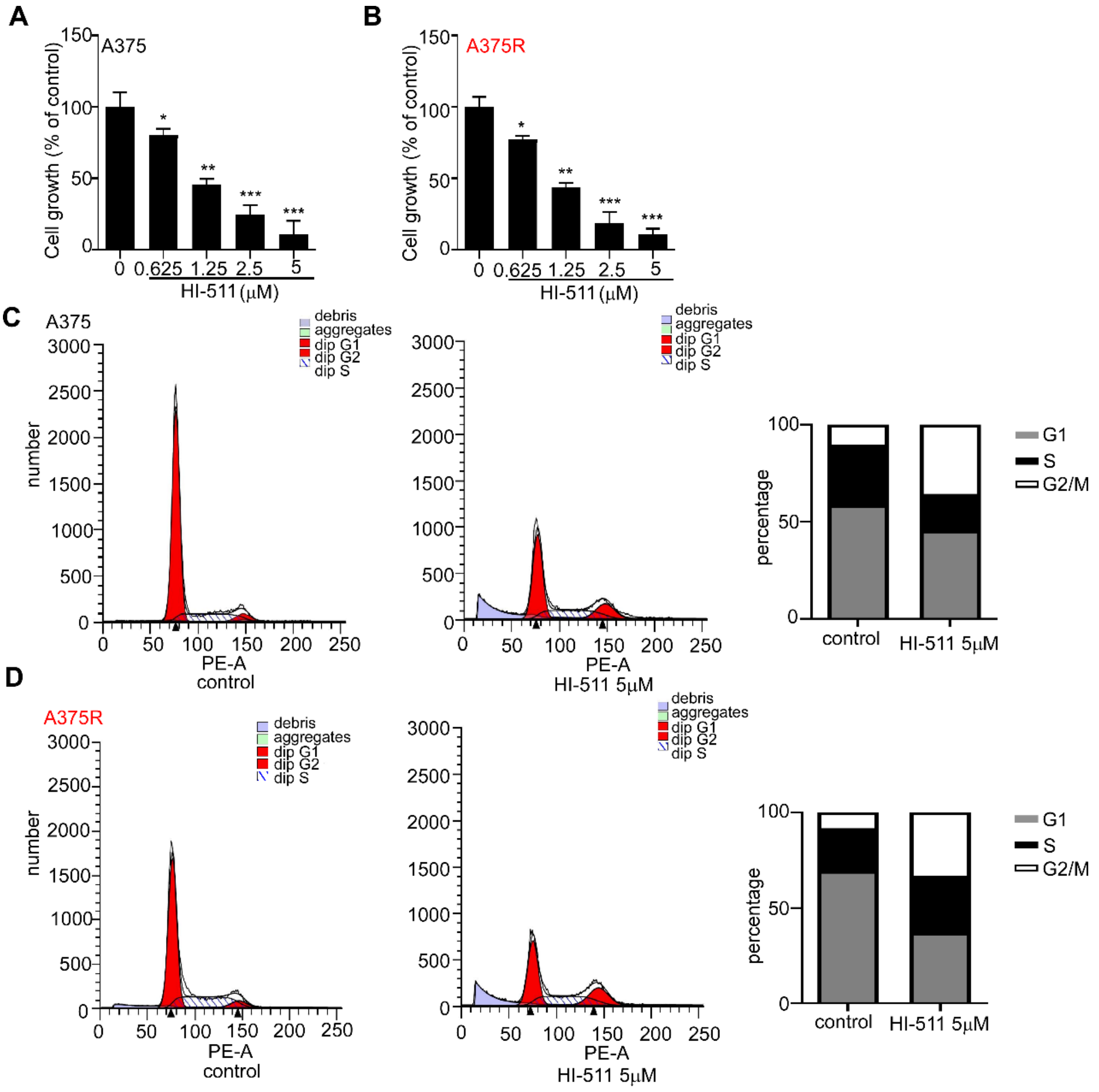
A
A375
Cell growth (% of control)
150
100
50
0
*
**
***
***
0
0.625
1.25
2.5
5
HI-511 (μM)
B
A375R
Cell growth (% of control)
150
100
50
0
*
**
***
***
0
0.625
1.25
2.5
5
HI-511 (μM)
C
A375
number
debris
aggregates
dip G1
dip G2
dip S
3000
2500
2000
1500
1000
500
0
0
50
100
150
200
250
PE-A
control
HI-511 5μM
percentage
100
50
0
G1
S
G2/M
control
HI-511 5μM
D
A375R
number
debris
aggregates
dip G1
dip G2
dip S
PE-A
control
HI-511 5μM
percentage
G1
S
G2/M
control
HI-511 5μM

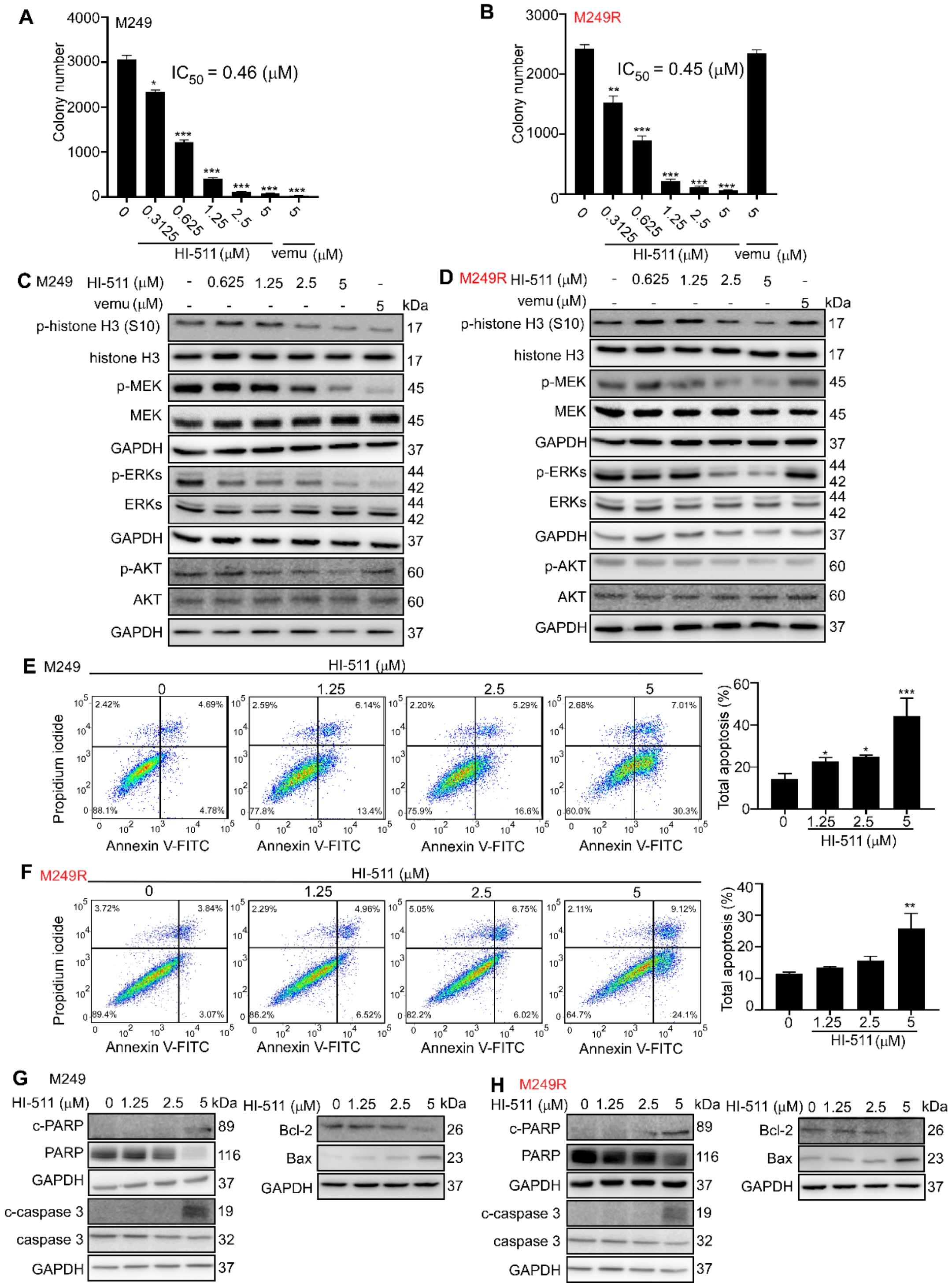

A
M249
Colony number
IC50 = 0.46 (μM)
HI-511 (μM)
vemu (μM)
B
M249R
Colony number
IC50 = 0.45 (μM)
HI-511 (μM)
vemu (μM)
C M249 HI-511 (μM) - 0.625 1.25 2.5 5 -
vemu (μM) - - - - - 5 kDa
p-histone H3 (S10)
histone H3
p-MEK
MEK
GAPDH
p-ERKs
ERKs
GAPDH
p-AKT
AKT
GAPDH
D M249R HI-511 (μM) - 0.625 1.25 2.5 5 -
vemu (μM) - - - - - 5 kDa
p-histone H3 (S10)
histone H3
p-MEK
MEK
GAPDH
p-ERKs
ERKs
GAPDH
p-AKT
AKT
GAPDH
E M249
HI-511 (μM)
Propidium iodide
Annexin V-FITC
Total apoptosis (%)
F M249R
HI-511 (μM)
Propidium iodide
Annexin V-FITC
Total apoptosis (%)
G M249
HI-511 (μM) 0 1.25 2.5 5 kDa
c-PARP
PARP
GAPDH
c-caspase 3
caspase 3
GAPDH
Bcl-2
Bax
GAPDH
H M249R
HI-511 (μM) 0 1.25 2.5 5 kDa
c-PARP
PARP
GAPDH
c-caspase 3
caspase 3
GAPDH
Bcl-2
Bax
GAPDH

A

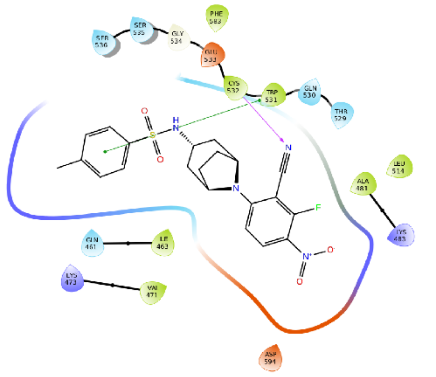

B

HI-511 binding with BRAF

BRAF binding residue	interaction	HI-511 binding area
Cys 532	H-bond	nitrile group
Trp 531	π-π stacking	phenyl ring

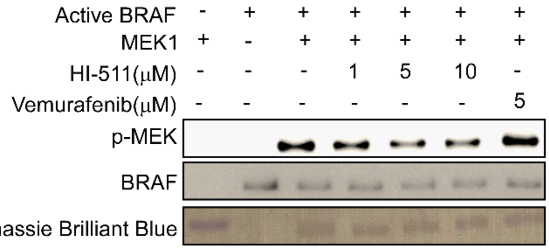

C

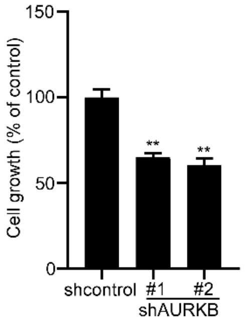

D

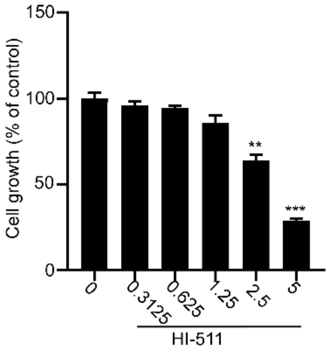

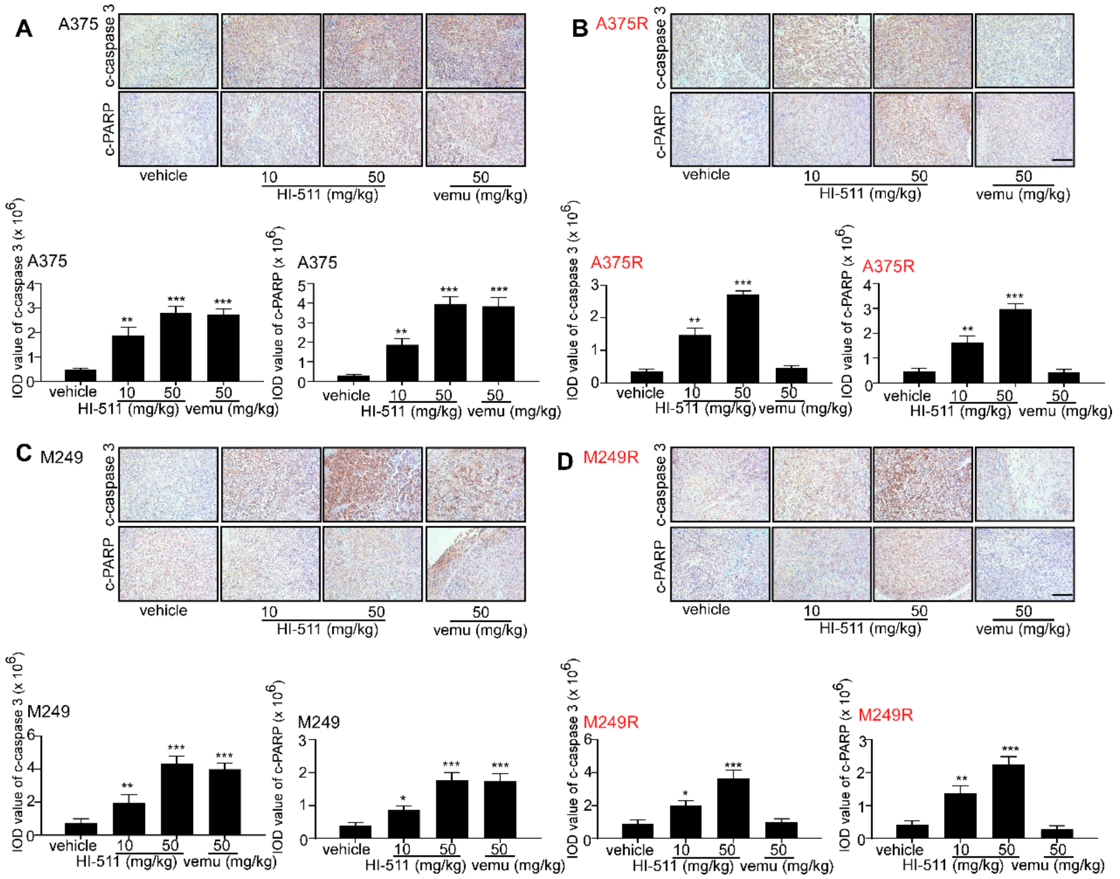
A
A375
c-caspase 3
c-PARP
vehicle
10
50
HI-511 (mg/kg)
50
vemu (mg/kg)
B
A375R
C
M249
D
M249R
IOD value of c-caspase 3 (x 10^6)
IOD value of c-PARP (x 10^6)
